# Systematic Review and Meta-analysis of Circulatory Disease from Exposure to Low-Level Ionizing Radiation and Estimates of Potential Population Mortality Risks

**DOI:** 10.1289/ehp.1204982

**Published:** 2012-06-22

**Authors:** Mark P. Little, Tamara V. Azizova, Dimitry Bazyka, Simon D. Bouffler, Elisabeth Cardis, Sergey Chekin, Vadim V. Chumak, Francis A. Cucinotta, Florent de Vathaire, Per Hall, John D. Harrison, Guido Hildebrandt, Victor Ivanov, Valeriy V. Kashcheev, Sergiy V. Klymenko, Michaela Kreuzer, Olivier Laurent, Kotaro Ozasa, Thierry Schneider, Soile Tapio, Andrew M. Taylor, Ioanna Tzoulaki, Wendy L. Vandoolaeghe, Richard Wakeford, Lydia B. Zablotska, Wei Zhang, Steven E. Lipshultz

**Affiliations:** 1 Radiation Epidemiology Branch, National Cancer Institute, National Institutes of Health, Department of Health and Human Services, Rockville, Maryland, USA; 2 Southern Urals Biophysics Institute, Ozyorsk, Russia; 3 Research Center for Radiation Medicine, Kyiv, Ukraine; 4 Health Protection Agency, Centre for Radiation, Chemical and Environmental Hazards, Chilton, United Kingdom; 5 Center for Research in Environmental Epidemiology (CREAL), Barcelona, Spain; 6 Medical Radiological Research Center of Russian Academy of Medical Sciences, Obninsk, Russia; 7 NASA Johnson Space Center, Space Radiation Program, Houston, Texas, USA; 8 Radiation Epidemiology Group, INSERM (Institut National de la Santé et de la Recherche Médicale) Unité U1018, Institut Gustave Roussy, Villejuif, France; 9 Department of Medical Epidemiology and Biostatistics, Karolinska Institute, Stockholm, Sweden; 10 Department of Radiotherapy and Radiation Oncology, University of Leipzig, Leipzig, Germany; 11 Department of Radiotherapy and Radiation Oncology, University of Rostock, Rostock, Germany; 12 Federal Office for Radiation Protection, Department of Radiation Protection and Health, Oberschleissheim, Germany; 13 Laboratoire d’Epidémiologie, Institut de Radioprotection et de Sûreté Nucleaire, Fontenay-aux-Roses, France; 14 Department of Epidemiology, Radiation Effects Research Foundation, Hiroshima City, Japan; 15 CEPN (Nuclear Evaluation Protection Center), Fontenay-aux-Roses, France; 16 Helmholtz Zentrum München, German Research Centre for Environmental Health, Institute of Radiation Biology (ISB), Radiation Proteomics, Oberschleissheim, Germany; 17 University College London Institute of Cardiovascular Sciences & Great Ormond Street Hospital for Children, London, United Kingdom; 18 Department of Epidemiology and Biostatistics, Imperial College Faculty of Medicine, London, United Kingdom; 19 Dalton Nuclear Institute, University of Manchester, Manchester, United Kingdom; 20 Department of Epidemiology and Biostatistics, University of California San Francisco School of Medicine, San Francisco, California, USA; 21 Department of Pediatrics, Leonard M. Miller School of Medicine, University of Miami, Miami, Florida, USA

**Keywords:** cancer, circulatory disease, heart disease, radiation, stroke

## Abstract

Background: Although high doses of ionizing radiation have long been linked to circulatory disease, evidence for an association at lower exposures remains controversial. However, recent analyses suggest excess relative risks at occupational exposure levels.

Objectives: We performed a systematic review and meta-analysis to summarize information on circulatory disease risks associated with moderate- and low-level whole-body ionizing radiation exposures.

Methods: We conducted PubMed/ISI Thomson searches of peer-reviewed papers published since 1990 using the terms “radiation” AND “heart” AND “disease,” OR “radiation” AND “stroke,” OR “radiation” AND “circulatory” AND “disease.” Radiation exposures had to be whole-body, with a cumulative mean dose of < 0.5 Sv, or at a low dose rate (< 10 mSv/day). We estimated population risks of circulatory disease from low-level radiation exposure using excess relative risk estimates from this meta-analysis and current mortality rates for nine major developed countries.

Results: Estimated excess population risks for all circulatory diseases combined ranged from 2.5%/Sv [95% confidence interval (CI): 0.8, 4.2] for France to 8.5%/Sv (95% CI: 4.0, 13.0) for Russia.

Conclusions: Our review supports an association between circulatory disease mortality and low and moderate doses of ionizing radiation. Our analysis was limited by heterogeneity among studies (particularly for noncardiac end points), the possibility of uncontrolled confounding in some occupational groups by lifestyle factors, and higher dose groups (> 0.5 Sv) generally driving the observed trends. If confirmed, our findings suggest that overall radiation-related mortality is about twice that currently estimated based on estimates for cancer end points alone (which range from 4.2% to 5.6%/Sv for these populations).

Based on observations in irradiated populations, the health risks of low-level exposure to ionizing radiation have been assumed to be related primarily to cancer. At high radiation doses a variety of other well-established effects are observed, in particular, damage to the structures of the heart and to the coronary, carotid, and other large arteries. This damage occurs both in patients receiving radiotherapy and in experimental animals (Adams et al. 2003). There are plausible, if not completely understood, mechanisms by which high doses of radiation affect the blood circulatory system (Schultz-Hector and Trott 2007). Recent analyses of the Japanese atomic-bomb survivors have suggested that excess mortality from noncancer disease was comparable to that from cancer (Ozasa et al. 2012; Preston et al. 2003).

An association between lower doses (< 0.5 Gy) and late circulatory disease has only recently been suspected and remains controversial. Recent reviews have presented evidence suggesting an excess radiation-induced risk at occupational and environmental dose levels [Advisory Group on Ionising Radiation (AGIR) 2010; Little et al. 2010]. In particular, a review by the Health Protection Agency’s AGIR in the United Kingdom estimated substantial excess risks for ischemic heart disease (IHD) and stroke, but concluded that a significantly elevated risk was detectable only for exposures above about 0.5 Gy (AGIR 2010). The AGIR report also reviewed biological data suggesting that many inflammatory end points potentially relevant to circulatory disease may be differentially regulated below and above about 0.5 Gy (AGIR 2010), emphasizing the importance of assessing risks associated with exposures of < 0.5 Gy.

Here, we test the hypothesis of a causal association between low-level radiation exposure and circulatory disease in a general unselected population. We estimate population circulatory disease mortality risks from low doses of radiation by extending recent meta-analyses (AGIR 2010; Little et al. 2008, 2009b, 2010) of Japanese atomic-bomb survivors and occupationally exposed groups, taking heterogeneity among studies into account. The results of the meta-analysis are used to estimate the potential radiation-related mortality risks of circulatory disease in various populations and to compare them with the risks of cancer.

## Data and Methods

*Data and meta-analysis.* Searches of the PubMed (U.S. National Library of Medicine, Washington, DC, USA) and ISI Thomson (Web of Knowledge, New York, New York, USA) databases were conducted on 14 May 2011 and 17 August 2011, respectively, using the terms “radiation” AND “heart” AND “disease,” OR “radiation” AND “stroke,” OR “radiation” AND “circulatory” AND “disease.” The ISI Thomson database search was restricted to human data. Only peer-reviewed papers from 1990 onward that had reliable ascertainment of circulatory disease morbidity or mortality were considered; abstracts and letters were not included. There was no restriction on the type of study design (e.g., cohort, case–control, case–base). Abstracts and papers were manually reviewed by M.P.L. and W.Z. A total of 4,971, 1,180, and 526 articles were published in PubMed in these categories since 1990; the ISI Thomson search (which was conducted using all three groups of search words combined) returned a total of 1,480 articles. Although there was no restriction to publication in English, based on assessment of the titles and abstracts the only studies meeting our criteria were published in that language.

Studies were excluded if there was no analysis of circulatory disease in relation to individual exposures or if there was not a reliable (e.g., film-badge or area-monitoring based) estimate of whole-body dose. All of the studies included in the analysis expressed radiation dose in sieverts (Sv), which should be very similar to unweighted absorbed doses in gray (Gy) [International Commission on Radiological Protection (ICRP) 2007]. Exposures had to involve moderate- or low-dose (cumulative mean < 0.5 Sv) whole-body exposure, or exposures at a low dose rate (i.e., < 10 mSv/day) and so included studies of environmental exposures, occupational exposures, or exposures experienced by Japanese atomic-bomb survivors. The reason for emphasizing uniform whole-body exposure is that the target tissue for radiation-associated circulatory disease is not known, thus whole-body dose [which will be approximately the same as dose to any tissue (ICRP 2007)] is the most reliable metric with which to compare studies. However, we also included two occupationally exposed groups with some degree of nonuniformity in exposure (e.g., in relation to liver, lung, and bone dose), although with uniform dose to the circulatory system (Azizova et al. 2010a, 2010b; Kreuzer et al. 2006). The requirement for uniform whole-body dose and analysis of circulatory disease in relation to individual dose resulted in the exclusion of a number of otherwise eligible studies, for example, the Massachusetts tuberculosis fluoroscopy cohort (Davis et al. 1989).

We excluded studies of any cohort in which the additional follow-up amounted to ≤ 1 year with respect to the larger analysis in which it is included. Therefore, we excluded U.S. and Canadian nuclear worker studies (Howe et al. 2004; Zablotska et al. 2004) that had no more follow-up (to 31 December 1997 and to 31 December 1994, respectively) than the International Agency for Research on Cancer (IARC) 15-country study (Vrijheid et al. 2007) that subsumed them. We also excluded the Canadian National Dose Registry study (Zielinski et al. 2009) that overlaps with the Canadian nuclear worker data (Zablotska et al. 2004) and has a somewhat lower quality of linkage to employment records and verification of dosimetry (Gilbert 2001) as well as a study by Atkinson et al. (2004) subsumed within the latest National Registry for Radiation Workers analysis cohort (Muirhead et al. 2009) and with earlier final follow-up (end 1997 vs. end 2001). Recent analyses of circulatory and related end points in the Japanese atomic-bomb survivor cohort that were published after our literature search were also not included (Adams et al. 2012; Ozasa et al. 2012; Takahashi et al. 2011, 2012); the mortality study of Ozasa et al. (2012) had identical follow-up (1950–2003) to an earlier paper by Shimizu et al. (2010) that was included in our analysis.

Having derived the primary study populations, we further selected studies so as to be more or less disjoint. We therefore did not include the study of Richardson and Wing (1999) because it is largely subsumed in the IARC 15-country study of Vrijheid et al. (2007), with minimal extra years of follow-up [to 31 December 1990 for Richardson and Wing (1999) vs. 31 December 1984 for Vrijheid et al. (2007)]. Likewise, we did not include the study of McGeoghegan et al. (2008) because the British Nuclear Fuels Limited worker cohort is largely subsumed within the study of Muirhead et al. (2009) and has only 4 more years of follow-up [to 31 December 2005 vs. 31 December 2001 for Muirhead et al. (2009)]. However, we tested for the effect of including both these studies in the meta-analysis.

Outcomes included in our analysis {generally coded to the *International Classification of Diseases, 10th Revision* [ICD-10; World Health Organization (WHO) 1992]} had to fall within one of the four major subtypes of circulatory disease determined *a priori*: ischemic heart disease (IHD, ICD-10 I20–I25); heart disease apart from IHD (non-IHD; ICD-10 I26–I52); cerebrovascular disease (CVA; ICD-10 I60–I69); and all other circulatory diseases (ICD-10 I00–I19, I53–I59, I70–I99). This resulted in the exclusion of the Talbott et al. (2003) study, which assessed only heart disease and so cannot be included within any of these four disease end points. For each study, we selected disjoint end-point groups with maximum coverage within these four circulatory disease subtype groups. We used morbidity rather than mortality data from the Mayak worker studies of Azizova et al. (2010a, 2010b) because of the significant loss of follow-up for the mortality study and low diagnostic accuracy for death certificate reporting for this cohort.

The results of the PubMed and ISI Thomson searches were cross-checked by M.P.L. and W.Z. Additional checks were made using ISI Thomson citations of various review articles (Little et al. 2008; McGale and Darby 2005) and other sources as detailed in Little et al. (2008). Meta-analysis Of Observational Studies in Epidemiology (MOOSE) group guidelines for meta-analysis were used (Stroup et al. 2000) [see Supplemental Material, Table S1 (http://dx.doi.org/10.1289/ehp.1204982) for a checklist indicating compliance with MOOSE guidelines].

A total of 10 studies met our criteria for inclusion. Although the Japanese data (Shimizu et al. 2010; Yamada et al. 2004) and many of the occupational studies included individuals with cumulative absorbed dose ranges of > 0.5 Sv, mean cumulative whole-body doses from external sources of radiation in cohorts included in our analysis were generally < 0.2 Sv [with the exception of the Mayak worker study, which had a mean dose of about 0.8 Gy (Azizova et al. 2010a, 2010b)], and the occupational cohorts were all exposed at low daily dose rates (generally < 1 mSv/day, and all < 10 mSv/day). Details regarding the quality of dosimetry, assessment of disease end points, selection criteria to determine cohort eligibility, circulatory disease risk factors assessed, and statistical analyses used in the 10 studies are provided in Supplemental Material, Table S2 (http://dx.doi.org/10.1289/ehp.1204982).

*Statistical methods for meta-analysis.* The analytical techniques extend those employed previously (AGIR 2010; Little et al. 2008, 2009b, 2010) to analyze different data (including studies of medically exposed populations as well as the studies included in this analysis). Pooled excess relative risk (ERR) per sievert were estimated for the four circulatory disease subgroups defined above.

In the absence of significant heterogeneity, we computed the best linear unbiased estimate (inverse-variance weighted) of ERR (*ERR_tot_*) as


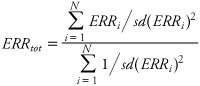
[1]

where *ERR_i_* indicates the ERR reported in the *i*th study. This estimate has an SD given by


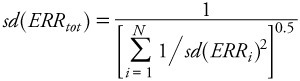
[2]

These formulae were used to compute aggregate measures of ERR and their associated 95% confidence intervals [obtained as *ERR*_tot_ ± *N*_0.975_ × *sd*(*ERR*_tot_)] in Table 2. (*N*_0.975_ ≈ 1.96 is the 97.5th percentile point of the standard normal distribution.) One-sided *p*-values were computed from the centiles of the normal distribution. Equation 2 provides a consistent estimate of the SD. SDs were estimated for the individual studies based on confidence intervals reported in the published papers.

**Table 1:** Estimated ERRs of circulatory disease in the Japanese atomic-bomb survivors and occupational and environmental exposure studies.

Data	Reference	Mean heart/brain dose (range) (Sv)	No. in cohort (person-years follow-up)	End point (mortality)^a^	ERR/Sv (95% CI)
Japanese atomic-bomb survivors					
Mortality	Shimizu et al. 2010	0.1 (0 to 4)b	86,611 (NA)	IHD (ICD-9 410–414)	0.02 (–0.10, 0.15)
				Rheumatic heart disease (ICD-9 393–398)	0.86 (0.25, 1.72)
				Heart failure (ICD-9 428)	0.22 (0.07, 0.39)
				Other heart disease (ICD-9 390–392, 415–427, 429)	–0.01 (–0.21, 0.24)
				CVA total (ICD-9 430–438)c	0.12 (0.05, 0.19)c
				Circulatory disease apart from heart disease and stroke (ICD-9 390–392, 401, 403, 405, 439–459)c	0.58 (0.45, 0.72)c
Morbidity	Yamada et al. 2004	0.1 (0 to 4)d	10,339 (NA)	Hypertension incidence, 1958–1998 (ICD-9 401)	0.05 (–0.01, 0.10)d
				Hypertensive heart disease incidence, 1958–1998 (ICD-9 402, 404)	–0.01 (–0.09, 0.09)d
				IHD incidence, 1958–1998 (ICD-9 410–414)	0.05 (–0.05, 0.16)d
				Aortic aneurysm incidence, 1958–1998 (ICD-9 441, 442)	0.02 (–0.22, 0.41)d
				CVA incidence, 1958–1998 (ICD-9 430, 431, 433, 434, 436)	0.07 (–0.08, 0.24)d
Occupational studies					
Mayak workers	Azizova et al. 2010a, 2010b	0.83 (0 to 5.92)e	12,210 (205,249)	IHD morbidity (ICD-9 410–414)	0.119 (0.051, 0.186)e,f
			12,210 (249,530)	CVA morbidity (ICD-9 430–432, 434, 436)	0.449 (0.338, 0.559)e,f
Chernobyl emergency workers	Ivanov et al. 2006	0.109 (0 to > 0.5)	61,017 (NA)	Hypertension (ICD-10 I10–I15) morbidity	0.26 (–0.04, 0.56)
				IHD (ICD-10 I20–I25) morbidity	0.41 (0.05, 0.78)
				Other heart disease (ICD-10 I30–I52) morbidity	–0.26 (–0.81, 0.28)
				CVA (ICD-10 I60–I69) morbidity	0.45 (0.11, 0.80)
				Morbidity from diseases of arteries, arterioles, and capillaries (ICD-10 I70–I79)	0.47 (–0.15, 1.09)
				Morbidity from diseases of veins, lymphatic vessels, and lymph nodes (ICD-10 I80–I89)	–0.26 (–0.70, 0.18)
German uranium miner study	Kreuzer et al. 2006	0.041 (0 to 0.909)e	59,001 (1,801,626)	CVA (ICD-10 I60–I69)	0.09 (–0.6, 0.8)e
EdF workers	Laurent et al. 2010	0.0215 (0 to 0.6)	22,393 (440,984)	IHD (ICD-10 I20–I25)	4.1 (–2.9, 13.7)g
				CVA (ICD-10 I60–I69)	17.4 (0.2, 43.9)g
Eldorado uranium miners and processing (male) workers	Lane et al. 2010	0.0522 (< 0.0234 to > 0.1215)	16,236 (508,673)	IHD (ICD-10 I20–I25)	0.15 (–0.14, 0.58)
				CVA (ICD-10 I60–I69)	–0.29 (< –0.29, 0.27)
Third analysis of UK National Registry for Radiation Workers	Muirhead et al. 2009	0.0249 (< 0.01 to > 0.4)	174,541 (3,900,000)	IHD (ICD-9 410–414)	0.259 (–0.05, 0.61)
				CVA (ICD-9 430–438)	0.161 (–0.42, 0.91)
IARC 15-country nuclear worker study	Vrijheid et al. 2007	0.0207 (0.0 to > 0.5)	275,312 (4,067,861)	IHD (ICD-10 I20–I25)	–0.01 (–0.59, 0.69)
				Heart failure (ICD-10 I50)	–0.03 (< 0, 4.91)
				CVA (ICD-10 I60–I69)	0.88 (–0.67, 3.16)
NA, not available. All data are in relation to underlying cause of death, unless otherwise indicated. Adapted from Little et al. (2008, 2010). aCoded to the International Classification of Diseases, 9th Revision (ICD-9; WHO 1977) or to ICD-10 (WHO 1992). bAnalysis based on colon dose. cAnalysis using underlying or contributing cause of death. dAnalysis based on stomach dose, derived from Table 3 of Yamada et al. (2004) with smoking and drinking in the stratification. eRisk estimates in relation to cumulative whole body external gamma dose. fAssuming a lag period of 10 years. g90% CI.					

Heterogeneity was assessed via the standard χ^2^ statistic and calculated as


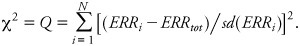
[3]

The above estimates correspond to a fixed-effect model, in which *ERR_i_* ~ *N*(μ, σ*_i_*^2^). When heterogeneity is statistically significant (assessed by comparing *Q* with centiles of the chi-square distribution with the appropriate number of degrees of freedom = *N* – 1) a random-effects model is more appropriate, in which we assume *ERR_i_* | δ*_i_* ~ *N*(μ *+* δ*_i_*,σ*_i_*^2^) and that δ*_i_ ~ N*(0,Δ^2^). The random-effects model assumes that inference is being made about a hypothetical population of studies of which the observed studies involved are assumed to constitute a “random sample” of potential studies of the same effects. Following DerSimonian and Laird (1986), we computed the 1-step estimate of Δ^2^ by equating the statistic *Q* and its expectation under this model to obtain


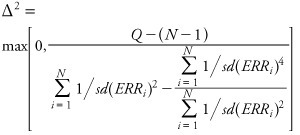
[4]

Similarly to the above, we then computed the best linear unbiased estimate (inverse-variance weighted) of ERR, given by


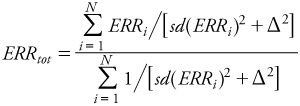
[5]

Similarly to the above, this estimate has an SD given by


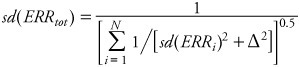
_[6]_

We estimated 1-sided *p*-values (assuming only detrimental effects) in the standard way from the mean, μ, and SD, σ, derived from the meta-analysis for each circulatory disease end point, as *p*[*N*(0,1) < –μ*/*σ]. Statistical significance was defined by *p <* 0.05. The Egger test of publication/selection bias (Egger et al. 1997; Steichen 1998) and the Duval and Tweedie (2000) “trim-and-fill” method of correction for publication/selection bias were employed, as shown in Supplemental Material, Table S3 (http://dx.doi.org/10.1289/ehp.1204982). All statistical models were fitted using Stata/SE 11.2 for Windows (32 bit) (StataCorp, College Station, TX).

*Estimates of population risks.* We used pooled ERR from the meta-analysis to derive population-based excess absolute risk (EAR) estimates according to underlying cause-specific mortality rates for each population. Specifically, we used estimates for the year 2003 in England and Wales (Office for National Statistics 2004), 2009 for Japan (Statistics and Information Department 2011), and the latest available WHO (2010) data for the following countries: China, for 2000; France, 2007; Germany, 2006; Russia, 2006; Spain, 2005; Ukraine, 2008; and the United States, 2005. We assumed a 5-year minimum latency period, after which the ERR was assumed to apply for the remainder of life. For all of the countries listed above, we estimated the risk of exposure-induced death (REID) per sievert, years of life lost per sievert, and years of life lost per radiation-induced circulatory disease death, by applying methods previously used to derive comparable estimates for radiation-induced cancer [United Nations Scientific Committee on the Effects of Atomic Radiation (UNSCEAR) 2008]. In addition, we obtained population risk estimates for radiation-induced solid cancers (ICD-10 C00–C80) and leukemias excluding chronic lymphocytic leukemia (ICD-10 C91–C95, excluding C91.1) for China, Japan, the United Kingdom, and the United States for comparison with population risk estimates for circulatory diseases (UNSCEAR 2008).

## Results

**Figure 1:**
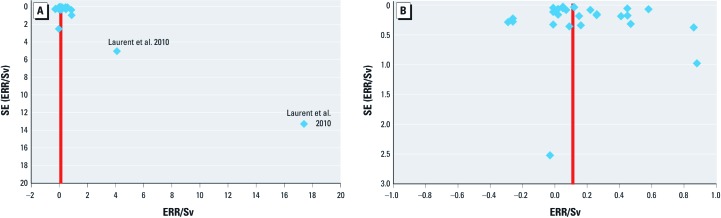
Funnel plot of ERR/Sv versus SE of ERR. Each circulatory disease end point comprising each of the four main circulatory disease subtypes (IHD, non-IHD, CVA, all circulatory disease apart from heart disease and stroke) for each study considered in the meta-analysis is plotted separately (*A*). The red line shows the aggregate random-effects ERR estimate. (*B*) Data excluding the study of Laurent et al. (2010).

*Meta-analysis*. A funnel plot shows little evidence of publication or selection bias in the meta-analysis—at least once the very large (but imprecise) ERRs in one study (Laurent et al. 2010) are removed (Figure 1). More formally, an Egger test for bias (Egger et al. 1997) revealed no significant evidence for publication or selection bias in any circulatory disease end point: Egger test *p*-values ranged from 0.322 for IHD to 0.692 for CVA, and little difference was made to risk coefficients if trim-and-fill publication/selection-bias correction methods were used (Duval and Tweedie 2000) [see Supplemental Material, Table S3 (http://dx.doi.org/10.1289/ehp.1204982)].

Table 1 demonstrates that most ERR estimates (22 of 29) are positive, and with the exception of the study of Laurent et al. (2010) are generally of modest size, with absolute values of < 1/Sv. The results of the meta-analysis (Table 2) using a random-effects model show a statistically significant ERR per sievert for IHD [ERR = 0.10/Sv, 95% confidence interval (CI): 0.04, 0.15, 1-sided *p <* 0.001], CVA (ERR = 0.21/Sv, 95% CI: 0.02, 0.39, 1-sided *p* = 0.014), and circulatory disease apart from heart disease and stroke (ERR = 0.19/Sv, 95% CI: –0.00, 0.38, 1-sided *p* = 0.026; –0.00 indicates that the number is between –0.005 and 0). The ERR for non-IHD is significant at least for the fixed-effect model (ERR = 0.12/Sv, 95% CI: –0.01, 0.25, 1-sided *p* = 0.031), but not for the random-effects model (ERR = 0.08/Sv, 95% CI: –0.12, 0.28, 1-sided *p* = 0.222) (Table 2). The heterogeneity in ERR between the various studies and end points for IHD and non-IHD is not statistically significant (*p* > 0.1), although it is significant for the other end points (*p* ≤ 0.001; Table 2).

**Table 2:** ERR coefficients for circulatory diseases as a result of exposure to low-level radiation ≥ 5 years earlier, by disease.

Disease	References	Fixed-effect estimate of ERR/Sv (95% CI)	Random-effect estimate of ERR/Sv (95% CI)	1-sided significance, *p*-value (fixed effect/random effect)	Heterogeneity χ^2^ (df)/*p*-value
IHD (ICD-10 I20–I25)	Azizova et al. 2010aa, Ivanov et al. 2006, Lane et al. 2010, Laurent et al. 2010, Muirhead et al. 2009, Shimizu et al. 2010, Vrijheid et al. 2007, Yamada et al. 2004	0.10 (0.05, 0.15)	0.10 (0.04, 0.15)	< 0.001/< 0.001	7.20 (7)/0.408
Non-IHD (ICD-10 I26–I52)	Ivanov et al. 2006, Shimizu et al. 2010b, Vrijheid et al. 2007c	0.12 (–0.01, 0.25)	0.08 (–0.12, 0.28)	0.031/0.222	4.65 (3)/0.199
CVA (ICD-10 I60–I69)	Azizova et al. 2010bd, Ivanov et al. 2006, Kreuzer et al. 2006, Lane et al. 2010, Laurent et al. 2010, Muirhead et al. 2009, Shimizu et al. 2010, Vrijheid et al. 2007, Yamada et al. 2004	0.20 (0.14, 0.25)	0.21 (0.02, 0.39)	< 0.001/0.014	34.28 (8)/< 0.001
Circulatory disease apart from heart disease and CVA (ICD-10 I00–I19, I53–I59, I70–I99)	Ivanov et al. 2006e, Shimizu et al. 2010f, Yamada et al. 2004g	0.10 (0.05, 0.14)	0.19 (–0.00, 0.38)	< 0.001/0.026	66.83 (7)/< 0.001
Values are from Table 1, unless otherwise indicated. aAnalysis based on morbidity from IHD, with a 10-year lag. bAnalysis based on mortality from heart failure and other heart disease. cAnalysis based on mortality from heart failure. dAnalysis based on morbidity from CVA, with a 10-year lag. eAnalysis based on morbidity from hypertension, disease of arteries, arterioles and capillaries, veins, lymphatic vessels, and lymph nodes. fAnalysis based on mortality from rheumatic heart disease and circulatory disease apart from heart disease and CVA. gAnalysis based on morbidity from hypertension, hypertensive heart disease, and aortic aneurysm.										

In general, ERR estimates were not particularly sensitive to the removal of individual studies [see Supplemental Material, Table S4, (http://dx.doi.org/10.1289/ehp.1204982)], though effects were greater for the end points addressed by only a few studies, in particular non-IHD (three studies) and all circulatory disease apart from heart disease and CVA (three studies). Exclusion of the Mayak workforce studies (Azizova et al. 2010a, 2010b) had the greatest effect, resulting in a random-effect ERR for IHD of 0.07 (95% CI: –0.01, 0.15) compared with 0.10 (95% CI: 0.04, 0.15) and 0.12 (95% CI: 0.02, 0.23) for CVA compared with 0.21 (95% CI: 0.02, 0.39). The addition of the Richardson and Wing (1999) or the McGeoghegan et al. (2008) data to the IHD category (the only circulatory disease group to which they can contribute) makes very little difference: The fixed-effects ERR changes from 0.10 (95% CI: 0.05, 0.15) (Table 2) to 0.10 (95% CI: 0.06, 0.15) or 0.10 (95% CI: 0.05, 0.15), respectively, and the random-effects ERR changes from 0.10 (95% CI: 0.04, 0.15) (Table 2) to 0.13 (95% CI: 0.04, 0.23) or 0.09 (95% CI: 0.03, 0.16), respectively.

*Population risks*. Population-based EAR estimates for REID for all circulatory disease range from 2.50%/Sv (CI: 0.77, 4.22) for France to 8.51%/Sv (95% CI: 4.00, 13.02) for Russia, reflecting the underlying risk of circulatory disease mortality (Table 3). Estimated circulatory disease mortality risks are generally dominated (in Germany, Russia, Ukraine, the United Kingdom, and the United States) by IHD and CVA (Tables 3 and 4). The random-effects model, based on aggregate ERR data from individual studies without age-at-exposure information, predicts that population circulatory disease EAR (i.e., REID) in the United Kingdom varies minimally with age at exposure (Table 5). However, in this instance more weight should be attached to models fitted to the current Japanese atomic-bomb survivor mortality data of Shimizu et al. (2010) [see Supplemental Material, Tables S5 and S6 (http://dx.doi.org/10.1289/ehp.1204982)], which provides information on variation of risk by age at exposure: risks reduce from 20.73%/Sv at ≤ 9 years of age to 2.05%/Sv at ≥ 70 years of age (Table 5)]. In this review, we found indications of the same direction of trend with age at exposure also in the French nuclear workers (Laurent et al. 2010), although there are no such trends (but apparently little power to assess them) in the IARC study (Vrijheid et al. 2007) (results not shown).

**Table 3:** Estimated EAR of REID for various subtypes of circulatory disease, by country.

Country (year underlying mortality rates were determined)	Baseline proportion of deaths due to circulatory disease (%)	REID × 10^–2^/Sv (95% CI)													
		IHD (ICD-10 I20–I25)^a^	Non-IHD (ICD-10 I26–I52)^a^	CVA (ICD-10 I60–I69)^a^	Other circulatory disease (ICD-10 I00–I19, I53–I59, I70–I99)^a^	All circulatory disease (ICD-10 I00–I99)^b^	Cancer risks			
							All solid cancer (ICD-10 C00–C80)		Leukemia excluding CLL (ICD-10 C91–C95, except C91.1)	
China (2000)	42.1	0.92 (0.41, 1.42)	0.11 (–0.16, 0.37)	4.31 (0.48, 8.14)	1.43 (–0.01, 2.86)	6.76 (2.63, 10.89)	3.95c	3.89d	0.27e	0.42f
France (2007)	20.8	0.50 (0.22, 0.78)	0.54 (–0.85, 1.94)	0.92 (0.10, 1.74)	0.53 (–0.00, 1.05)	2.50 (0.77, 4.22)	—		—	
Germany (2006)	48.7	1.71 (0.76, 2.65)	0.97 (–1.52, 3.46)	1.69 (0.19, 3.19)	1.38 (–0.01, 2.76)	5.75 (2.39, 9.10)	—		—	
Japan (2009)	31.1	0.57 (0.25, 0.88)	0.80 (–1.25, 2.85)	2.19 (0.24, 4.14)	0.45 (–0.00, 0.91)	4.01 (1.13, 6.89)	4.65c	4.90d	0.32e	0.43f
Russia (2006)	64.4	2.82 (1.26, 4.39)	0.31 (–0.49, 1.11)	4.59 (0.51, 8.66)	0.79 (–0.00, 1.57)	8.51 (4.00, 13.02)	—		—	
Spain (2005)	35.8	0.91 (0.41, 1.42)	0.82 (–1.28, 2.52)	1.91 (0.21, 3.60)	0.81 (–0.00, 1.63)	4.45 (1.73, 7.17)
Ukraine (2008)	69.2	4.14 (1.85, 6.43)	0.20 (–0.31, 0.70)	2.85 (0.31, 5.39)	0.93 (–0.00, 1.85)	8.11 (4.53, 11.69)
United Kingdom (2003)	39.9	1.70 (0.76, 2.64)	0.37 (–0.58, 1.32)	2.24 (0.25, 4.22)	0.76 (–0.00, 1.53)	5.07 (2.55, 7.58)	5.15c	4.40d	0.38e	0.43f
United States (2005)	39.3	1.82 (0.81, 2.82)	0.57 (–0.89, 2.03)	1.29 (0.14, 2.44)	0.80 (–0.00, 1.61)	4.48 (2.22, 6.74)	4.74c	4.41d	0.47e	0.42f
CLL, chronic lymphocytic leukemia. All calculations assume a single acutely delivered test dose of 0.01 Sv, and are calculated assuming a random-effects model. Cancer data are from UNSCEAR (2008). aRelative risk coefficients for IHD, non-IHD, CVA, and all circulatory disease apart from heart disease and CVA are from Table 2. bObtained by summing the risks from component disease categories (IHD, non-IHD, CVA, and other circulatory). cRelative risk model with linear-quadratic dose response, adjusted for sex, age, and years since exposure. dAdditive risk model with linear-quadratic dose response, adjusted for age and years since exposure. eRelative risk model with linear-quadratic dose response, adjusted for age. fAdditive risk model with linear-quadratic dose response, adjusted for sex and years since exposure.																	

**Table 4:** Estimated population mortality risks for subtypes of circulatory disease and cancer in the United Kingdom.

Disease	REID × 10–2/Sv (95% CI)	Years of life lost/Sv (95% CI)	Years of life lost/ radiation-induced death (95% CI)
IHD (ICD-10 I20–I25)a	1.70 (0.76, 2.64)	0.146 (0.065, 0.227)	8.61 (8.61, 8.61)
Non-IHD (ICD-10 I26–I52)a	0.37 (–0.58, 1.32)	0.027 (–0.043, 0.097)	7.36 (7.36, 7.36)
CVA (ICD-10 I60–I69)a	2.24 (0.25, 4.22)	0.162 (0.018, 0.307)	7.26 (7.26, 7.26)
Other circulatory disease (ICD-10 I00–I19, I53–I59, I70–I99)a	0.76 (–0.00, 1.53)	0.065 (–0.000, 0.130)	8.50 (8.50, 8.50)
All circulatory disease (ICD-10 I00–I99)b	5.07 (2.55, 7.58)	0.400 (0.209, 0.591)	7.90 (7.90, 7.90)
Solid cancerc	5.15	0.711	13.8
Solid cancerd	4.40	0.632	14.4
Leukemiae	0.38	0.075	19.8
Leukemiaf	0.43	0.135	31.6
All calculations assume a single acutely delivered test dose of 0.01 Sv, and are calculated assuming a random-effects model. aRelative risk coefficients for IHD, non-IHD, CVA, and all circulatory disease apart from heart disease and CVA are from Table 2. bObtained by summing the risks from component disease categories (IHD, non-IHD, CVA, and other circulatory). cRelative risk model with linear-quadratic dose response, adjusted for sex, age, and years since exposure (taken from UNSCEAR 2008). dAdditive risk model with linear-quadratic dose response, adjusted for age and years since exposure (taken from UNSCEAR 2008). eRelative risk model with linear-quadratic dose response, adjusted for age (taken from UNSCEAR 2008). fAdditive risk model with linear-quadratic dose response, adjusted for sex and years since exposure (taken from UNSCEAR 2008).						

**Table 5:** Variation of population mortality risks of circulatory disease and cancer with age at exposure in the United Kingdom.

Age at exposure, years	Circulatory disease							Cancer														
	LSS model with adjustment for age at exposure			Meta-analysis without adjustment for age at exposure																		
							Solid cancer							Leukemia						
	REID × 10^–2^/Sv	Years of life lost/Sv	REID × 10^–2^/Sv (95% CI)^a^	Years of life lost/Sv (95% CI)^a^	REID × 10^–2^/Sv			Years of life lost/Sv			REID × 10^–2^/Sv			Years of life lost/Sv		
0–9	20.73	1.836	5.25 (2.67, 7.83)	0.459 (0.242, 0.676)	11.07b,c	8.36c,d	1.798b,c	1.412c,d	0.74c,e	0.70c,f	0.270c,e	0.335c,f
10–19	14.18	1.260	5.26 (2.68, 7.84)	0.459 (0.242, 0.676)	9.19b,c	7.39c,d	1.371b,c	1.199c,d	0.52c,e	0.65c,f	0.118c,e	0.269c,f
20–29	10.09	0.898	5.27 (2.69, 7.86)	0.458 (0.242, 0.674)	7.45b,c	6.34c,d	1.042b,c	0.966c,d	0.46c,e	0.59c,f	0.080c,e	0.208c,f
30–39	7.48	0.661	5.29 (2.69, 7.89)	0.453 (0.240, 0.667)	5.77b,c	5.20c,d	0.742b,c	0.722c,d	0.43c,e	0.53c,f	0.065c,e	0.153c,f
40–49	5.75	0.494	5.30 (2.70, 7.90)	0.439 (0.232, 0.646)	4.15b,c	4.01c,d	0.475b,c	0.486c,d	0.40c,e	0.46c,f	0.053c,e	0.105c,f
50–59	4.53	0.364	5.30 (2.68, 7.91)	0.410 (0.215, 0.606)	2.68b,c	2.83c,d	0.259b,c	0.284c,d	0.37c,e	0.38c,f	0.042c,e	0.065c,f
60–69	3.57	0.249	5.19 (2.59, 7.80)	0.355 (0.181, 0.528)	1.48b,c	1.75c,d	0.113b,c	0.136c,d	0.31c,e	0.29c,f	0.029c,e	0.035c,f
≥ 70	2.05	0.107	3.90 (1.83, 5.96)	0.200 (0.095, 0.305)	0.45b,c	0.66c,d	0.025b,c	0.036c,d	0.17c,e	0.16c,f	0.011c,e	0.011c,f
All age	8.53	0.732	5.07 (2.55, 7.58)	0.400 (0.209, 0.591)	5.15b	4.40d	0.711b	0.632d	0.38e	0.43f	0.075e	0.135f
All calculations assume a single acutely delivered test dose of 0.01 Sv (unless otherwise indicated), and are calculated assuming a random-effects model. Cancer data are from UNSCEAR (2008). The Life Span Study (LSS) predictions given in columns 2, 3 are based on the optimal model (model 5) fitted to the data of Shimizu et al. (2010) shown in Supplemental Material, Table S6 (http://dx.doi.org/10.1289/ehp.1204982). aObtained by summing the risks from component disease categories (IHD, non-IHD, CVA, and other circulatory). bRelative risk model with linear-quadratic dose response, adjusted for sex, age, and years since exposure. cSingle acutely delivered test dose of 0.1 Sv. dAdditive risk model with linear-quadratic dose response, adjusted for age and years since exposure. eRelative risk model with linear-quadratic dose response, adjusted for age. fAdditive risk model with linear-quadratic dose response, adjusted for sex and years since exposure.																								

In aggregate, EAR coefficients are similar to those for cancer mortality, and the indications are that, as for cancer, there is a pronounced reduction of risk with increasing age at exposure (Table 5); for example, UNSCEAR (2008) estimated that the total cancer REID is in the range 4.16–5.58% for China, Japan, the United Kingdom, and the United States (Table 3). In different terms, the risks for a UK population are 0.146 (95% CI: 0.065, 0.227) years of life lost per sievert and 8.61 years of life lost per radiation-induced death, and 0.162 years of life lost per sievert (95% CI: 0.018, 0.307) and 7.26 years of life lost per radiation-induced death, for IHD and CVA, respectively (Table 4). These years of life lost per radiation-induced death figures are substantially lower than the corresponding ones for solid cancers (13.8–14.4 years) and leukemia (19.8–31.6 years; Table 4), reflecting the fact that circulatory disease mortality tends to occur later in life.

## Discussion

We estimated statistically significant ERRs for four subtypes of circulatory disease in persons exposed to radiation. There was significant heterogeneity among individual study estimates for CVA and other circulatory diseases, but not for IHD and non-IHD. These results confirm and extend a previous analysis that also found statistically significant ERRs for IHD and CVA (AGIR 2010).

Most of the studies considered in the present review involved low-to-moderate mean cumulative radiation doses (≤ 0.2 Gy), with participants in the occupational studies exposed at near-background dose rates. Nevertheless, the small numbers of participants exposed at high cumulative doses (≥ 0.5 Gy) drive the observed trends in most cohorts with these higher dose groups (Table 1).

Population-based EAR estimates for circulatory disease mortality were dominated by estimated risks for IHD and CVA, which is unsurprising, given that deaths from these two end points account for the largest number of deaths from circulatory disease and that the excess risk is a simple multiple of the underlying circulatory disease risk.

A critical question in these calculations is whether the risk coefficients derived here are applicable to the lower cumulative doses (< 100 mSv) or low dose rates (< 5 mSv/hr) of principal relevance to radiological protection. We fitted a linear ERR model to the data in the meta-analysis, so we implicitly assumed a linear association of risk at low doses and dose rates. There is little evidence for nonlinearity in the dose–response curve for circulatory disease in Japanese atomic-bomb survivors (Shimizu et al. 2010; Yamada et al. 2004) or in the Mayak workers (Azizova et al. 2010a, 2010b), so this assumption seems reasonable in the current analysis. At least for IHD and non-IHD, additional support for a linear relationship between risk and low doses or low dose rates can be derived from the consistency of ERR per sievert between Japanese atomic-bomb survivors with moderate radiation doses at high dose rates (Shimizu et al. 2010; Yamada et al. 2004) and occupational cohorts with protracted exposures. Currently, an etiologic mechanism for associations between low-level radiation and circulatory disease risk is unclear, so there are no sound biological grounds on which to base selection of a model for extrapolating the risks to low doses or low dose rates (AGIR 2010). However, a candidate mechanism, based on monocyte cell killing in the intima, suggests that circulatory disease risks would be approximately proportional to dose at low dose rates (Little et al. 2009a), but because of saturation of repair systems, effects would be greater for exposures to higher doses and dose rates (UNSCEAR 1993). Although this mechanism is consistent with the occupational data, it is speculative and not yet experimentally confirmed. Epidemiological data suggest that circulatory disease risk is significantly elevated only for acute or cumulative doses of about 0.5 Gy and above; nonetheless, the dose rate independence of risk remains (AGIR 2010).

All studies included in the meta-analysis were either of the Japanese atomic-bomb survivors or of occupationally exposed groups. All occupational groups were to some extent selected, from populations that were sufficiently fit to be employed as radiation workers. The degree of selection (as a result of mortality in the period from the bombings in August 1945 to the assembly of the cohort in October 1950) in the Japanese atomic-bomb survivor cohort has long been controversial (Little and Charles 1990; Stewart and Kneale 1984). There is evidence of selection in at least the earlier years of follow-up for some noncancer end points (Ozasa et al. 2012; Preston et al. 2003). As risks in a general unselected population are likely to be higher than in a selected one, it is possible that the risks given here underestimate those that are applicable to a general population; they are more likely to be correct for occupationally exposed groups subject to a similar degree of healthy-worker selection as those considered here.

We estimated ERR, the metric used in most published data (AGIR 2010). Accordingly, for the population risk estimates, we assumed a relative risk model for projecting risk to the end of life, starting 5 years after exposure. ERR does not substantially vary by sex or time since exposure in Japanese atomic-bomb survivors (Little 2004; Preston et al. 2003; Shimizu et al. 2010), although there is variation by age at exposure [see Supplemental Material, Tables S5, S6 (http://dx.doi.org/10.1289/ehp.1204982)], and increasing time-since-exposure trends have been observed in other groups (Vrijheid et al. 2007). Implicitly, we also assumed that ERR is invariant across populations. This assumption may be reasonable for IHD and non-IHD ERRs, which did not show statistically significant heterogeneity across exposed populations (Japanese atomic-bomb survivors and largely European/American occupational data), but this assumption may not be appropriate for the other circulatory disease subgroups, where heterogeneity was significant.

Candidate biological mechanisms for the effects of radiation on circulatory disease have been recently reviewed (AGIR 2010; Little et al. 2008; Schultz-Hector and Trott 2007). At high radiotherapeutic doses (> 5 Gy), the cell-killing effect on capillaries and endothelial cells plausibly explains effects on the heart and other parts of the circulatory system (Schultz-Hector and Trott 2007). At lower doses (i.e., 0.5–5 Gy), human data and *in vivo* and *in vitro* experiments have demonstrated that many inflammatory markers are up-regulated long after exposure to radiation, although for exposures less than about 0.5 Gy the balance shifts toward anti-inflammatory effects (Little et al. 2008; Mitchel et al. 2011), implying that the initiating mechanisms for adverse effects in this dose range would not directly result from inflammation. A recent analysis of renal failure mortality in the atomic-bomb survivors suggests that radiation-induced renal dysfunction may be a factor in causing increased circulatory disease (Adams et al. 2012).

The generally uniform whole-body, low linear energy transfer radiation in the cohorts we analysed is uninformative as to specific target tissues. What the target tissues are for circulatory system effects at moderate and low doses (< 0.5 Gy) remains uncertain. Dose-related variations in T-cell and B-cell populations in Japanese atomic-bomb survivors suggest that the immune system may be adversely affected (Kusunoki et al. 1998). Together with the known involvement of the immune system in cardiovascular disease (Danesh et al. 2002; Ridker 1998; Whincup et al. 2000), these results suggest that whole-body or bone-marrow dose might be the most relevant to radiation effects. A mechanism based on monocyte cell killing in the arterial intima suggests that the target for atherosclerosis is the arterial intima (Little et al. 2009a); however, as noted above, this mechanism remains speculative.

In their reviews, Little et al. (2008, 2010) have documented abundant radiobiological reasons for considering studies of moderate and low doses separately from studies of high (i.e., radiotherapeutic) doses because mechanisms of effect are likely to differ. That said, the risks observed in radiotherapeutic studies [see Supplemental Material, Table S7 (http://dx.doi.org/10.1289/ehp.1204982)] are not inconsistent with those in the lower-dose studies that are the focus of the present review and suggest common mechanisms over this dose range. However, given the modest level of excess risk at these lower doses, and the many lifestyle factors that can affect the risk of circulatory disease, attributing causation to the observed associations requires caution. Interpreting the results of studies in which there is no, or at best limited, lifestyle information, that is to say in studies apart from the Japanese atomic-bomb survivors (Shimizu et al. 2010; Yamada et al. 2004) and the Mayak nuclear workers (Azizova et al. 2010a, 2010b), would be particularly speculative.

The substantial and statistically significant heterogeneity in the estimated relative risks of circulatory disease apart from heart disease among the studies considered is not surprising given variation in the distributions of different risk factors across populations, but it limits interpretation of the observed associations for these end points. Epidemiological research has identified specific risk factors for circulatory disease, including male sex, family history of heart disease, cigarette smoking, diabetes, high blood pressure, obesity, increased low-density lipoprotein cholesterol, and decreased high-density lipoprotein cholesterol plasma levels (Burns 2003; Wilson et al. 1998). Lifestyle factors (in particular, shift work in occupational groups) (Tüchsen et al. 2006) and infections (Danesh et al. 2002; Ridker 1998; Whincup et al. 2000) are also potential risk factors for circulatory disease. We could not correct for any of these variables in our meta-analysis. Statistical methods (i.e., random-effects models) are available to accommodate heterogeneity (DerSimonian and Laird 1986), but these methods may not adequately account for the variation induced by confounding or effect modification. The interactions of these risk factors with possible radiation effects are unknown, but confounding or effect modification cannot be ruled out in studies in which no adjustment was made; in the two cohorts where it was possible to make adjustment for such risk factors little difference was made to radiation risk (Azizova et al. 2010a, 2010b; Shimizu et al. 2010).

A potential problem in meta-analyses is publication bias, which selects against studies that do not produce significant findings, potentially biasing pooled estimates upwards, or selection bias on the part of those selecting the cohorts from the database searches, which could be either positive or negative. We believe that publication bias is unlikely because radiation-induced cardiovascular disease has been an issue in the Japanese atomic-bomb survivor data for at least 15 years (Preston et al. 2003; Shimizu et al. 1992; Wong et al. 1993); in consequence, such negative findings are likely to be of sufficient interest to be published, and therefore this should not greatly affect the findings of our meta-analysis, concentrating as it does on results published since 1990. There is little internal evidence of either publication or selection bias [Figure 1; see also Supplemental Material, Table S3 (http://dx.doi.org/10.1289/ehp.1204982)], although at least for the end points of non-IHD and all other circulatory disease, the Egger test has little power. The fact that the two persons (M.P.L., W.Z.) evaluating the database search agreed on the included studies also suggests that selection bias is minimal.

We chose to limit our results to studies published as full papers and referenced in PubMed or ISI Thomson. We judged that the most important and high quality studies are likely to be published as full papers. All of the studies selected were cohort studies (although this was not a criterion for being chosen), and all had reasonable quality dosimetry [see Supplemental Material, Table S2 (http://dx.doi.org/10.1289/ehp.1204982)]. Only two of the studies, those of the Japanese atomic-bomb survivors (Shimizu et al. 2010) and of Mayak workers (Azizova et al. 2010a, 2010b), had information on lifestyle factors, in particular cigarette smoking, drinking, and other variables associated with circulatory disease. The lack of evidence of strong positive associations between various nonmalignant smoking-related respiratory diseases and dose in various worker studies (Laurent et al. 2010; Muirhead et al. 2009; Vrijheid et al. 2007) suggests that cigarette smoking is unlikely to have been an important positive confounder of the association with circulatory disease in these groups, and that bias will therefore be if anything towards the null. Information on socioeconomic status (industrial vs. nonindustrial, educational level) in various worker studies (Laurent et al. 2010; McGeoghegan et al. 2008; Muirhead et al. 2009; Vrijheid et al. 2007) provides only partial control for confounding by lifestyle/environmental risk factors.

Although we eliminated studies with a large degree of overlap, some degree of overlap remained among studies included in the meta-analysis, particularly for the morbidity and mortality data for the Japanese atomic-bomb survivors (Shimizu et al. 2010; Yamada et al. 2004). However, the largest component of circulatory disease morbidity, hypertension (about half the total number of cases), has a much lower ERR, 0.05/Sv (Yamada et al. 2004), than either CVA, 0.12/Sv, or heart disease, 0.18/Sv, mortality (Shimizu et al. 2010), suggesting that the overlap may not be large. There is also likely to be statistical dependence between the risks of some end points within the atomic-bomb survivor morbidity study (Yamada et al. 2004), although in the most likely overlapping categories (hypertension, hypertensive heart disease, CVA), the numbers involved are relatively modest. The effect of removing the morbidity study (Yamada et al. 2004) from the analysis [see Supplemental Material, Table S4 (http://dx.doi.org/10.1289/ehp.1204982)] is generally to slightly increase risks; there is a more substantial elevation for circulatory disease apart from heart disease and CVA, but this contributes relatively modestly (6–25%) to overall circulatory disease mortality (Table 3). There is overlap between the UK worker study (Muirhead et al. 2009) and the 15-country worker study (Vrijheid et al. 2007), but this is probably not substantial because the former has 9 more years of follow-up (1993–2001) and the latter includes data from 14 countries in addition to the United Kingdom.

Some of the heterogeneity that we observed in relation to circulatory disease apart from heart disease is driven by morbidity versus mortality differences, reinforcing previous findings (Little et al. 2010). Although one can argue that relative risks should not be different for mortality and morbidity (although absolute risks very well could be), the varying definitions and ascertainment of morbidity end points mean that different degrees of severity of circulatory disease are being encompassed. The relative risks of mortality data should be more similar (than mortality vs. morbidty) (Little et al. 2010), although the uncertainty from misclassification remains and varies over time. Both outcome and exposure misclassification would be expected to bias results toward the null in most cases, unless the bias was differential (e.g., outcome misclassification associated with exposure) (Copeland et al. 1977). We used morbidity and mortality data in the Japanese atomic-bomb survivors, which contribute to some extent independently (as discussed above) and are of similar quality (Shimizu et al. 2010; Yamada et al. 2004). However, we used morbidity rather than mortality data in the Mayak worker studies (Azizova et al. 2010a, 2010b) because of the significant problems with the loss of follow-up in the mortality data (which occurred as soon as workers moved out of the closed cities in the ex-USSR) and the much lower diagnostic accuracy in this cohort of death certificate reporting.

In the Japanese atomic-bomb survivors, respiratory and digestive diseases were also elevated (Ozasa et al. 2012; Preston et al. 2003), implying a lack of specificity of risk in this cohort. However, there is no evidence of excess risk for any nonmalignant diseases apart from circulatory disease in the other cohorts considered here (Laurent et al. 2010; Muirhead et al. 2009; Vrijheid et al. 2007).

## Conclusions

Our meta-analysis supports an association between low doses and low dose rates of ionizing radiation and an excess risk of IHD. For non-IHD, the association is statistically significant when using (as is justifiable, given the homogeneity of risk) a fixed-effect model. The association is less certain for other circulatory diseases given the heterogeneity in these end points among the studies. The evidence presented in this review indicates a need to conduct more detailed epidemiological studies that are capable of addressing potential confounding and misclassifying factors and possible selection bias that could influence these results as well as a particular need for a better understanding of biological mechanisms that might be responsible for the association. The estimates of population-based excess mortality risks for circulatory disease are similar to those for radiation-induced cancer, as also noted previously in relation to noncancer disease (Preston et al. 2003). If associations between low-level exposure to radiation and circulatory diseases reflect an underlying causal relationship that is linear at low doses, then the overall excess risk of mortality after exposure to low doses or low dose rates of radiation may be about twice that currently assumed based on estimated risks of mortality due to radiation-induced cancers alone.

## Supplemental Material

(348 KB) PDF

## References

[r1] Adams MJ, Grant EJ, Kodama K, Shimizu Y, Kasagi F, Suyama A (2012). Radiation dose associated with renal failure mortality: a potential pathway to partially explain increased cardiovascular disease mortality observed after whole-body irradiation.. Radiat Res.

[r2] Adams MJ, Hardenbergh PH, Constine LS, Lipshultz SE (2003). Radiation-associated cardiovascular disease.. Crit Rev Oncol Hematol.

[r3] AGIR (Advisory Group on Ionising Radiation) (2010). Circulatory Disease Risk. Report of the Independent Advisory Group on Ionising Radiation. London:Health Protection Agency.. http://www.hpa.org.uk/webc/HPAwebFile/HPAweb_C/1284475204588.

[r4] Atkinson WD, Law DV, Bromley KJ, Inskip HM (2004). Mortality of employees of the United Kingdom Atomic Energy Authority, 1946–97.. Occup Environ Med.

[r5] Azizova TV, Muirhead CR, Druzhinina MB, Grigoryeva ES, Vlasenko EV, Sumina MV (2010a). Cardiovascular diseases in the cohort of workers first employed at Mayak PA in 1948–1958.. Radiat Res.

[r6] Azizova TV, Muirhead CR, Druzhinina MB, Grigoryeva ES, Vlasenko EV, Sumina MV (2010b). Cerebrovascular diseases in the cohort of workers first employed at Mayak PA in 1948–1958.. Radiat Res.

[r7] Burns DM (2003). Epidemiology of smoking-induced cardiovascular disease.. Prog Cardiovasc Dis.

[r8] Copeland KT, Checkoway H, McMichael AJ, Holbrook RH (1977). Bias due to misclassification in the estimation of relative risk.. Am J Epidemiol.

[r9] Danesh J, Whincup P, Lewington S, Walker M, Lennon L, Thomson A (2002). *Chlamydia pneumoniae* IgA titres and coronary heart disease: prospective study and meta-analysis.. Eur Heart J.

[r10] Davis FG, Boice JD, Hrubec Z, Monson RR (1989). Cancer mortality in a radiation-exposed cohort of Massachusetts tuberculosis patients.. Cancer Res.

[r11] DerSimonian R, Laird N (1986). Meta-analysis in clinical trials.. Control Clin Trials.

[r12] Duval S, Tweedie R (2000). A nonparametric “trim and fill” method of accounting for publication bias in meta-analysis.. J Am Stat Assoc.

[r13] Egger M, Davey SG, Schneider M, Minder C (1997). Bias in meta-analysis detected by a simple, graphical test.. BMJ.

[r14] Gilbert ES (2001). Invited commentary: studies of workers exposed to low doses of radiation.. Am J Epidemiol.

[r15] Howe GR, Zablotska LB, Fix JJ, Egel J, Buchanan J (2004). Analysis of the mortality experience amongst U.S. nuclear power industry workers after chronic low-dose exposure to ionizing radiation.. Radiat Res.

[r16] ICRP (International Commission on Radiological Protection) (2007). The 2007 Recommendations of the International Commission on Radiological Protection. ICRP Publication 103.. Ann ICRP.

[r17] Ivanov VK, Maksioutov MA, Chekin SY, Petrov AV, Biryukov AP, Kruglova ZG (2006). The risk of radiation-induced cerebrovascular disease in Chernobyl emergency workers.. Health Phys.

[r18] Kreuzer M, Kreisheimer M, Kandel M, Schnelzer M, Tschense A, Grosche B (2006). Mortality from cardiovascular diseases in the German uranium miners cohort study, 1946–1998.. Radiat Environ Biophys.

[r19] Kusunoki Y, Kyoizumi S, Hirai Y, Suzuki T, Nakashima E, Kodama K (1998). Flow cytometry measurements of subsets of T, B and NK cells in peripheral blood lymphocytes of atomic bomb survivors.. Radiat Res.

[r20] Lane RS, Frost SE, Howe GR, Zablotska LB (2010). Mortality (1950–1999) and cancer incidence (1969–1999) in the cohort of Eldorado uranium workers.. Radiat Res.

[r21] Laurent O, Metz-Flamant C, Rogel A, Hubert D, Riedel A, Garcier Y (2010). Relationship between occupational exposure to ionizing radiation and mortality at the French electricity company, period 1961–2003.. Int Arch Occup Environ Health.

[r22] Little MP (2004). Threshold and other departures from linear-quadratic curvature in the non-cancer mortality dose–response curve in the Japanese atomic bomb survivors.. Radiat Environ Biophys.

[r23] Little MP, Charles MW (1990). Bomb survivor selection and consequences for estimates of population cancer risks.. Health Phys.

[r24] Little MP Gola A Tzoulaki I 2009a A model of cardiovascular disease giving a plausible mechanism for the effect of fractionated low-dose ionizing radiation exposure. PLoS Comput Biol 5 e1000539 doi:10.1371/journal.pcbi.1000539 [Online 23 October 2009] 19851450 PMC2759077

[r25] Little MP, Tawn EJ, Tzoulaki I, Wakeford R, Hildebrandt G, Paris F (2008). A systematic review of epidemiological associations between low and moderate doses of ionizing radiation and late cardiovascular effects, and their possible mechanisms.. Radiat Res.

[r26] Little MP, Tawn EJ, Tzoulaki I, Wakeford R, Hildebrandt G, Paris F (2010). Review and meta-analysis of epidemiological associations between low/moderate doses of ionizing radiation and circulatory disease risks, and their possible mechanisms.. Radiat Environ Biophys.

[r27] Little MP, Tawn EJ, Tzoulaki I, Wakeford R, Hildebrandt G, Tapio S (2009b). Comments: The non-cancer mortality experience of male workers at British Nuclear Fuels plc, 1946–2005.. Int J Epidemiol.

[r28] McGale P, Darby SC (2005). Low doses of ionizing radiation and circulatory diseases: a systematic review of the published epidemiological evidence.. Radiat Res.

[r29] McGeoghegan D, Binks K, Gillies M, Jones S, Whaley S (2008). The non-cancer mortality experience of male workers at British Nuclear Fuels plc, 1946–2005.. Int J Epidemiol.

[r30] Mitchel RE, Hasu M, Bugden M, Wyatt H, Little MP, Gola A (2011). Low-dose radiation exposure and atherosclerosis in *ApoE*^-/-^ mice.. Radiat Res.

[r31] Muirhead CR, O’Hagan JA, Haylock RGE, Phillipson MA, Willcock T, Berridge GLC (2009). Mortality and cancer incidence following occupational radiation exposure: third analysis of the National Registry for Radiation Workers.. Br J Cancer.

[r32] Office for National Statistics (2004). Mortality Statistics: Cause. Series DH2 no. 30. Review of the Registrar General on Deaths by Cause, Sex and Age, in England and Wales, 2003.

[r33] Ozasa K, Shimizu Y, Suyama A, Kasagi F, Soda M, Grant EJ (2012). Studies of the mortality of atomic bomb survivors, report 14, 1950–2003: an overview of cancer and noncancer diseases.. Radiat Res.

[r34] Preston DL, Shimizu Y, Pierce DA, Suyama A, Mabuchi K (2003). Studies of mortality of atomic bomb survivors. Report 13: Solid cancer and noncancer disease mortality: 1950–1997.. Radiat Res.

[r35] Richardson DB, Wing S (1999). Radiation and mortality of workers at Oak Ridge National Laboratory: positive associations for doses received at older ages.. Environ Health Perspect.

[r36] Ridker PM (1998). Inflammation, infection, and cardiovascular risk: how good is the clinical evidence?. Circulation.

[r37] Schultz-Hector S, Trott KR (2007). Radiation-induced cardiovascular diseases: is the epidemiologic evidence compatible with the radiobiologic data?. Int J Radiat Oncol Biol Phys.

[r38] Shimizu Y, Kato H, Schull WJ, Hoel DG (1992). Studies of the mortality of A-bomb survivors. 9. Mortality, 1950–1985: Part 3. Noncancer mortality based on the revised doses (DS86).. Radiat Res.

[r39] Shimizu Y Kodama K Nishi N Kasagi F Suyama A Soda M 2010 Radiation exposure and circulatory disease risk: Hiroshima and Nagasaki atomic bomb survivor data, 1950–2003. BMJ 340 b5349 doi:10.1136/bmj.b5349 [Online 14 January 2010] 20075151 PMC2806940

[r40] Statistics and Information Department (2011). Vital Statistics of Japan, 2009.

[r41] Steichen TJ (1998). sbe19: Tests for publication bias in meta-analysis. Stata Technical Bulletin 41:9–15.

[r42] Stewart AM, Kneale GW (1984). Non-cancer effects of exposure to A-bomb radiation.. J Epidemiol Community Health.

[r43] Stroup DF, Berlin JA, Morton SC, Olkin I, Williamson GD, Rennie D (2000). Meta-analysis of observational studies in epidemiology: a proposal for reporting. Meta-analysis Of Observational Studies in Epidemiology (MOOSE) group.. JAMA.

[r44] Takahashi I Abbott RD Ohshita T Takahashi T Ozasa K Akahoshi M 2012 A prospective follow-up study of the association of radiation exposure with fatal and non-fatal stroke among atomic bomb survivors in Hiroshima and Nagasaki (1980–2003). BMJ Open 2 e000654 doi:10.1136/bmjopen-2011-000654 [Online 3 February 2012] PMC3274709 22307102

[r45] Takahashi I, Geyer SM, Nishi N, Ohshita T, Takahashi T, Akahoshi M (2011). Lifetime risk of stroke and impact of hypertension: estimates from the adult health study in Hiroshima and Nagasaki.. Hypertens Res.

[r46] Talbott EO, Youk AO, McHugh-Pemu KP, Zborowski JV (2003). Long-term follow-up of the residents of the Three Mile Island accident area: 1979–1998.. Environ Health Perspect.

[r47] Tüchsen F, Hannerz H, Burr H (2006). A 12 year prospective study of circulatory disease among Danish shift workers.. Occup Environ Med.

[r48] UNSCEAR (United Nations Scientific Committee on the Effects of Atomic Radiation) (1993). Sources and Effects of Ionizing Radiation. UNSCEAR 1993 Report to the General Assembly, with Scientific Annexes. New York:United Nations.. http://www.unscear.org/unscear/en/publications/1993.html.

[r49] UNSCEAR (United Nations Scientific Committee on the Effects of Atomic Radiation) (2008). UNSCEAR 2006 Report. Annex A. Epidemiological Studies of Radiation and Cancer.. New York:United Nations, 13–322.

[r50] Vrijheid M, Cardis E, Ashmore P, Auvinen A, Bae JM, Engels H (2007). Mortality from diseases other than cancer following low doses of ionizing radiation: results from the 15-Country Study of nuclear industry workers.. Int J Epidemiol.

[r51] Whincup P, Danesh J, Walker M, Lennon L, Thomson A, Appleby P (2000). Prospective study of potentially virulent strains of *Helicobacter pylori* and coronary heart disease in middle-aged men.. Circulation.

[r52] WHO (World Health Organization) (1977). Manual of the International Statistical Classification of Diseases, Injuries and Causes of Death. Ninth Revision.

[r53] WHO (World Health Organization) (1992). International Statistical Classification of Diseases and Related Health Problems. Tenth Revision.

[r54] WHO (World Health Organization) (2010). World Health Organization Statistical Information System (WHOSIS) (updated 1 July 2010).. http://www.who.int/whosis/mort/download/en/index.html.

[r55] Wilson PW, D’Agostino RB, Levy D, Belanger AM, Silbershatz H, Kannel WB (1998). Prediction of coronary heart disease using risk factor categories.. Circulation.

[r56] Wong FL, Yamada M, Sasaki H, Kodama K, Akiba S, Shimaoka K (1993). Noncancer disease incidence in the atomic bomb survivors: 1958–1986.. Radiat Res.

[r57] Yamada M, Wong FL, Fujiwara S, Akahoshi M, Suzuki G (2004). Noncancer disease incidence in atomic bomb survivors, 1958–1998.. Radiat Res.

[r58] Zablotska LB, Ashmore JP, Howe GR (2004). Analysis of mortality among Canadian nuclear power industry workers after chronic low-dose exposure to ionizing radiation.. Radiat Res.

[r59] Zielinski JM, Ashmore PJ, Band PR, Jiang H, Shilnikova NS, Tait VK (2009). Low dose ionizing radiation exposure and cardiovascular disease mortality: cohort study based on Canadian National Dose Registry of radiation workers.. Int J Occup Med Environ Health.

