# Supplementary material for: Systematic Review and Meta-analysis of Circulatory Disease from Exposure to Low-Level Ionizing Radiation and Estimates of Potential Population Mortality Risks
Source: Environ Health Perspect. 2012 Jun 22;120(11):1503–11. doi: 10.1289/ehp.1204982 (PMC3556625; doi:10.1289/ehp.1204982)
Supplement: (348 KB) PDF [file ehp.1204982.s001.pdf]

Supplemental Material

Systematic Review and Meta-Analysis of Circulatory Disease from Exposure to Low-Level Ionizing Radiation and Estimates of Potential Population Mortality Risks

Mark P. Little, Tamara V. Azizova, Dimitry Bazyka, Simon D. Bouffler, Elisabeth Cardis, Sergey Chekin, Vadim V. Chumak, Francis A. Cucinotta, Florent de Vathaire, Per Hall, John D. Harrison, Guido Hildebrandt, Victor Ivanov, Valeriy V. Kashcheev, Sergiy V. Klymenko, Michaela Kreuzer, Olivier Laurent, Kotaro Ozasa, Thierry Schneider, Soile Tapio, Andrew M. Taylor, Ioanna Tzoulaki, Wendy L. Vandoolaeghe, Richard Wakeford, Lydia B. Zablotska, Wei Zhang, Steven E. Lipshultz

Contents

p.3-4: Supplemental Material A. Modeling of circulatory disease mortality in the Japanese atomic-bomb survivors (Shimizu et al. 2010)

p. 5-8: Supplemental Material, Table S1. MOOSE meta-analysis checklist

p. 9-17: Supplemental Material, Table S2. Assessment of study quality, with scoring.

p.18: Supplemental Material, Table S3. Assessments of publication/selection bias using methods of Egger et al. (1997) and Steichen (1998), and bias-corrected ERR coefficients using method of Duval and Tweedie (2000).

p.19-21: Supplemental Material Table S4. Sensitivity of Risk Estimates to Study Exclusion

p.22: Supplemental Material, Table S5. Models fitted individually to stroke, heart disease and all other circulatory disease in the Japanese atomic-bomb survivor Life Span Study mortality data

p.23: **Supplemental Material, Table S6. Models jointly fitted to stroke, heart disease and all other circulatory disease in the Japanese atomic-bomb survivor Life Span Study mortality data (optimal model shown in boldface)**

p.24: **Supplemental Material, Table S7. Estimated Excess Relative Risks of Circulatory Disease in Radiotherapeutically Treated Groups.**

p.25-26: **Reference List.**

Supplemental Material A. Modeling of circulatory disease mortality in the Japanese atomic-bomb survivors (Shimizu et al. 2010)

Methods

The Life Span Study mortality data of Shimizu *et al.* (2010) involve the follow-up of 86,611 survivors of the atomic-bombings of Hiroshima and Nagasaki from 1 October 1950 to 31 December 2003. By the end of 2003, 19,045 survivors had died of circulatory disease, and there were 25,113 deaths in which circulatory disease was mentioned as a contributory or underlying cause of death on the death certificate. Data is only provided by the Radiation Effects Research Foundation (RERF) in freely downloadable form for the endpoints of: (a) stroke (ICD9 430-438); (b) heart disease (ICD9 393-400, 402, 404, 406-429); and (c) all other circulatory disease (ICD9 390-392, 401, 403, 405, 439-459). We shall concentrate in Supplemental Material A on analysis of deaths in which circulatory disease was mentioned as a contributory or underlying cause of death for these three endpoints.

The expected number of such deaths in the Japanese A-bomb survivor data in with disease endpoint i (i=stroke, heart disease, other circulatory disease), stratum j and dose group d with average age at exposure, e, years, average years since exposure, y, and colon dose, D, is assumed to be given by:

$$PY_{ijd}\lambda_{ij}\left[1+ERR_i D(1+\beta_i D)\exp[\alpha_i(e-30)+\beta_i(y-30)]\right] \qquad \text{(A1)}$$

The stratum-specific background rates λ_{ij} are estimated by model fitting. The model is fitted by Poisson maximum likelihood (McCullagh and Nelder 1989) separately for each disease endpoint (Supplemental Material, Table S5) and also simultaneously across all three disease endpoints (stroke, heart disease, other circulatory disease) (Supplemental Material, Table S6) in order to test for heterogeneity of effect. Equation (A1) describes a linear-quadratic relative risk model.

Results

There is a highly significant increasing dose response for all three endpoints (p<0.001) (Supplemental Material, Table S5). There is a statistically significant effect of sex (p=0.022) for heart disease (Supplemental Material, Table S5), but otherwise there is no significant modification of dose response by sex or time since exposure for any endpoint (p>0.05), nor is there any significant curvature in the dose response for any endpoint (p>0.1) (Supplemental Material, Table S5). For stroke and other circulatory disease there are statistically significant (p<0.01) reductions in relative risk with increasing age at exposure, although this is not the case for heart disease (p=0.391) (Supplemental Material, Table S5). As shown by Supplemental Material, Table S6, the main effect excess relative risk coefficients are highly statistically significantly different (p<0.001) for the three endpoints. There is marginally statistically significant (p=0.053) heterogeneity also in the magnitude of the reductions of relative risk with increasing age at exposure.

Table S1. MOOSE (Stroup et al. 2000) meta-analysis checklist.

Category	Met	Where met and described, or if not met, reasons why not
**Problem definition**	Yes	See Abstract “Objective” section.
**Hypothesis statement**	Yes	See Introduction para 3, sentence 1.
**Description of study outcome(s)**	Yes	See Abstract “Results”+“Conclusions” sections.
**Type of exposure or intervention used**	Yes	See Introduction para 3, sentence 1.
**Type of study designs used**	Yes	See “Data and methods/Data and meta-analysis” section para 1, sentence 4.
**Study population**	Yes	See Introduction para 3, sentence 1.
**Qualifications of searchers (e.g., librarians and investigators)**	Yes	MPL has a D.Phil. in mathematics, WZ has a Ph.D.
**Search strategy, including time period included in the synthesis and keywords**	Yes	See “Data and methods/Data and meta-analysis” section para 1, sentence 1: Medline/ISI Thompson search using the terms “radiation” + “heart” + “disease” or “radiation” + “stroke” or “radiation” + “circulatory” + “disease”; only peer reviewed papers from 1990
**Effort to include all available studies, including contact with authors**	Yes	Contact with the authors of all studies selected for the final analysis (as in Table 1) was attempted: however, the requested data proved to be unobtainable in all but three cases (Japanese LSS mortality, IARC 15-country, EdF workers).
**Databases and registries searched**	Yes	See “Data and methods/Data and meta-analysis” section para 1, sentence 1: Medline and ISI Thompson (Web of Knowledge) databases were used.
**Search software used, name and version, including special features used**	N/A	Hand search was used.
**Use of hand searching (e.g., reference lists of obtained articles)**	Yes	See “Data and methods/Data and meta-analysis” section para 1, sentence 5.
**List of citations located and those excluded, including justification**	No	Given the numbers of searched papers (>6500), it is impossible to justify the inclusion or exclusion of each study. Among those that met preliminary requirements a secondary exclusion based on study quality was used, that would possibly have excluded some studies, but this was not in fact the case.

Supplemental Material, Table S1 (cont.)

Category	Met	Where met and described, or if not met, reasons why not
**Method of addressing articles published in languages other than English**	Yes	See "Data and methods/Data and meta-analysis" section para 1, sentence 7. As we state there, "Although there was no restriction to publication in English, based on assessment of the titles and abstracts the only studies meeting our criteria were published in that language.".
**Method of handling abstracts and unpublished studies**	No	See "Data and methods/Data and meta-analysis" section para 1, sentence 3. As we state in the Discussion, para 12 "We chose to limit our results to studies published as full papers … We judge that the most important and high quality studies are likely to be published as full papers."
**Description of any contact with authors**	Yes	As above, contact with the authors of all studies used in the final analysis (as in Table 1) was attempted: however, the requested data proved to be unobtainable in all but three cases (Japanese LSS mortality, IARC 15 country, EdF workers).
**Description of relevance or appropriateness of studies assembled for assessing the hypothesis to be tested**	Yes	We discuss the appropriateness of the results of the paper to general unselected populations in Discussion para 5.
**Rationale for the selection and coding of data (e.g., sound clinical principles or convenience)**	Yes	The data used is the obvious epidemiological data to use for the assessment of circulatory disease risk.
**Documentation of how data were classified and coded (e.g., multiple raters, blinding and inter-rater reliability)**	Yes	There were two raters, MPL and WZ, assessing studies blind to each other. An objective scoring system was used (see Supplemental Material Table S2), so there was 100% agreement.
**Assessment of confounding (e.g., comparability of cases and controls in studies where appropriate)**	Yes	There is extensive discussion of the possibility of confounding, in particular by lifestyle factors, in the Discussion paras 9-10.
**Assessment of study quality, including blinding of quality assessors; stratification or regression on possible predictors of study results**	No	We do not regard the blinding of quality assessors as a sensible requirement. There are numerous clues within each publication as to the cohort being studied and the principal authors, so that blinding would be all but impossible. The small numbers of studies, and the limited information available limit the usefulness of meta-regression; in any case we do not regard this form of analysis as very sensible given the likelihood of ecological bias.

Supplemental Material, Table S1 (cont.)

Category	Met	Where met and described, or if not met, reasons why not
**Assessment of heterogeneity**	Yes	This was assessed at some length (using fixed- and random-effects models), as described in the Statistical Methods for Meta-Analysis section of the main paper.
**Description of statistical methods (e.g., complete description of fixed or random effects models, justification of whether the chosen models account for predictors of study results, dose-response models, or cumulative meta-analysis) in sufficient detail to be replicated**	Yes	The statistical methodology (fixed- and random-effects model etc) is described at some length in the Statistical Methods for Meta-Analysis section of the main paper.
**Provision of appropriate tables and graphics**	Yes	See Tables 1-5, Supplemental Material, Tables S1-S7 and Figure 1.
**Graphic summarising individual study estimates and overall estimate**	Yes	No (such information is given in Table 1).
**Table giving descriptive information for each study included**	Yes	See Table 1 and Supplemental Material, Table S2.
**Results of sensitivity testing (e.g., subgroup analysis)**	Yes	See Supplemental Material, Table S4.
**Indication of statistical uncertainty of findings**	Yes	See Tables 1-5, Supplemental Material, Tables S4-S5.
**Quantitative assessment of bias (e.g., publication bias)**	Yes	A descriptive funnel plot (see Figure 1) demonstrates adequately that there is little or no publication or selection bias. This was supplemented more formally by use of the publication/selection-bias test of Egger *et al.* and Steicher, and assessment of bias using the trim-and-fill method of Duval and Tweedie (Supplemental Material, Table S3), all of which also suggested little or no publication or selection bias.
**Justification of exclusion (e.g., exclusion of non-English language citations)**	Yes	"Data and methods/Data and meta-analysis" section para 1, sentence 3. As we state in the Discussion, para 12 "We chose to limit our results to studies published as full papers and referenced in Medline or ISI Thompson. We judge that the most important and high quality studies are likely to be published as full papers."
**Assessment of quality of included studies**	Yes	See Supplemental Material, Table S2.
**Consideration of alternative explanations for observed results**	Yes	There is extensive discussion of the possibility of confounding, in particular by lifestyle factors, in the Discussion paras 9-10.

Supplemental Material, Table S1 (cont.)

Category	Met	Where met and described, or if not met, reasons why not
**Generalization of the conclusions (i.e., appropriate for the data presented and within the domain of the literature review)**	Yes	There is extensive discussion of the generalization of the conclusions to unselected general populations, given the possibility of (a) confounding, in particular by lifestyle factors, and (b) selection in the study cohorts considered, in the Discussion paras 5, 9-10.
**Guidelines for future research**	Yes	See Discussion final para (16).
**Disclosure of funding source**	Yes	See the Acknowledgements.

Table S2. Assessment of study quality, with scoring[a].

Data	Reference	Quality of dosimetry	Endpoint (mortality *vs* morbidity etc)	Selection criteria	Lifestyle circulatory disease risk factors assessed	Statistical analysis	Overall quality score/comment
Life Span Study atomic-bomb survivor mortality	Shimizu et al. 2010	3/5: DS02 colon doses based on interviews generally 5-15 years after bombings, assuming a neutron relative biological effectiveness of 10. No attempt made to adjust for dose error.	4/5: Mortality via Japanese national mortality registers (koseki/honseki), with analyses both of underlying and contributing causes of death in the period 1/10/1950-31/12/2003. Virtually complete ascertainment.	5/5: The Life Span Study (LSS) cohort comprises all survivors identified on Japanese national census of 1/10/1950 and special surveys between 1950 and 1953 as being resident in Hiroshima and Nagasaki at the times of bombings (6/8/1945 (Hiroshima) and 9/8/1945 (Nagasaki)) and within 2.5 km of hypocentres, and sample of persons exposed between 2.5-10 km from hypocentres.	4/5: Adjustment for information from 36,468 respondents to postal survey mailed to 51,965 in 1978 on smoking (never, past, present < 20/day, present>20/day), alcohol intake (regular, seldom/never), education (primary or less, secondary, college/university), type of household occupation (professional/technical, clerical/sales, farmer/craftsmen, transportation/service), obesity (body mass index)(<20, 20-25, >25), and diabetes mellitus (yes, no).	4/5: Poisson regression linear in external radiation dose, with adjustment for city, sex, age at exposure, attained age and lifestyle variables	4.0/5: Possible bias in identified cause of death: survivors are known to physicians. Very complete lifestyle information for a subset of the cohort.

Supplemental Material, Table S2 (cont.)

Data	Reference	Quality of dosimetry	Endpoint (mortality *vs* morbidity etc)	Selection criteria	Lifestyle circulatory disease risk factors assessed	Statistical analysis	Overall quality score/comment
Adult Health Study atomic-bomb survivor morbidity	Yamada et al. 2004	4/5: DS86 stomach doses based on interviews generally 5-15 years after bombings, assuming a neutron relative biological effectiveness of 10 and adjusting for dose error.	2/5: Morbidity assessed via biennial health examination in the period 1/7/1958-30/6/1998. Date of disease incidence assessed as midpoint between intervals of examination.	5/5: A subset of Life Span Study cohort assembled in 1958. To be included in the analysis cohort, subjects must have attended at least two AHS examinations in the period 1/7/1958 – 30/6/1998.	1/5: Adjustment for information on alcohol consumption (drink currently, drank in the past, never drank) and cigarette smoking (smoke currently, smoked in the past, never smoked) derived from four Life Span Study mail surveys (1965, 1969-1970, 1979-1980, 1991).	4/5: Poisson regression linear in external radiation dose with adjustment for city, sex age at time of bombing, age at examination and calendar time, and for certain analyses cigarette smoking and alcohol consumption.	3.2/5: Arguably ascertaining less severe forms of circulatory disease than those that result in death. Possible inaccuracies in assigned date of disease incidence.

Supplemental Material, Table S2 (cont.)

Data	Reference	Quality of dosimetry	Endpoint (mortality *vs* morbidity etc)	Selection criteria	Lifestyle circulatory disease risk factors assessed	Statistical analysis	
Mayak workers	Azizova et al. 2010a; 2010b	3/5: Individual external exposures recorded via photographic film. External γ-ray doses were estimated using "Mayak Doses-2005". Neutron exposures were not assessed. No attempt made to adjust for dose error.	5/5: Morbidity assessed among workers and ex-workers still resident in Ozyorsk via examinations every three months during 1948-1954, every six months during 1955-1960 and annually from 1960. Review of all clinical data by team of experts.	5/5: Cohort defined by having first worked at Mayak in 1948-1958. Follow-up from start of operations in 1948 to 31/12/2000.	3/5: Adjustment for individual information on cigarette smoking (smoker, never smoker, unknown), alcohol consumption (drinker, never drinker, unknown), blood pressure and body mass index (BMI) were assessed from the health examinations.	5/5: Poisson regression linear in external radiation dose with adjustment (via stratification) for age, calendar period, period of first employment at the plant, type of plant (reactors, radiochemical, plutonium), internal plutonium dose and lifestyle variables. BMI and blood pressure were adjusted for at baseline, and cigarette smoking and drinking were adjusted at the last interview before the first diagnosis of circulatory disease. There was analysis of interaction with various variables, including gender and attained age.	4.2/5: Arguably ascertaining less severe forms of circulatory disease than those that result in death. Good quality ascertainment of morbidity, lifestyle information.

Supplemental Material, Table S2 (cont.)

Data	Reference	Quality of dosimetry	Endpoint (mortality *vs* morbidity etc)	Selection criteria	Lifestyle circulatory disease risk factors assessed	Statistical analysis	Overall quality score/comment
Chernobyl emergency workers	Ivanov et al. 2006	2/5: Individual accumulated doses assessed from individual film badge dosimeters, or from dose rates in homes and workplaces. No attempt made to adjust for dose error.	4/5: Morbidity assessed in period 1996-2000 via specialized examinations by regular doctors (examination interval unspecified). Cerebrovascular disease confirmed via specialized neurological departments.	5/5: Analysis restricted to males with known radiation dose living in the European part of Russian Federation (Northwest, North-Caucasus, Volgo-Vyatsky, Povolzhsky, Central-Chernozemny, Ural regions) and registered in the Russian National Medical and Dosimetric Registry (RNMDR) on or before 1/1/1992, with information on health status between working in Chernobyl and 2000, and excluding those with cardiovascular disease before work at Chernobyl.	0/5: Adjustment for region (between which circulatory disease morbidity rates differed).	4/5: Poisson regression linear in external radiation dose with adjustment for age, region (Northwest, North-Caucasus, Volgo-Vyatsky, Povolzhsky, Central-Chernozemny, Ural), year of arrival to Chernobyl exclusion zone.	3.0/5: Area- based dosimetry used in some cases a weakness. Lack of detail as to clinical data used.

Supplemental Material, Table S2 (cont.)

Data	Reference	Quality of dosimetry	Endpoint (mortality *vs* morbidity etc)	Selection criteria	Lifestyle circulatory disease risk factors assessed	Statistical analysis	Overall quality score/comment
German uranium miner study	Kreuzer et al. 2006	2/5: External γ-ray exposure assessment via detailed job-exposure matrix for each calendar year, job type, etc.. based on measurements of γ-rays from 1955 onwards – estimates before this were based on the first available measurements, with adjustment for uranium content etc. No attempt made to adjust for dose error.	4/5: Mortality assessed from start of operations in 1946 to 31/12/1998.	5/5: Stratified random sample of 64,311 workers selected by time period of first work at Wismut (1946-1954, 1955-1970, 1971-1989), subject to being male and having worked at least 6 months at Wismut, date of employment between 1946-1989, year of birth after 1899.	0/5: None.	4/5: Poisson regression linear in external radiation dose with adjustment by attained age and calendar period, doses lagged by 5 years.	3.0/5: Dosimetry via job-exposure matrix a weakness, although as errors are likely to be Berkson this should not introduce bias. Absence of lifestyle information.

Supplemental Material, Table S2 (cont.)

Data	Reference	Quality of dosimetry	Endpoint (mortality *vs* morbidity etc)	Selection criteria	Lifestyle circulatory disease risk factors assessed	Statistical analysis	Overall quality score/comment
EdF workers	Laurent et al. 2010	4/5: Individual external exposures recorded via photographic film and assessments made of colon dose. Some correction for photon dose error. Neutron dose was not included, other than as a categorical adjusting variable (those with neutron dose > 10% of photon dose) in the analysis.	4/5: Mortality follow-up to 31/12/2003	5/5: All EdF workers who had worked at least 1 year for the company and who had been monitored for ionizing radiation exposure between 1961 and 1994. Follow-up started at later of date of initial employment + 1 year, 1/1/1968 or start of radiation monitoring.	1/5: Stratification by educational level at hiring.	5/5: Poisson regression linear in external radiation dose with adjustment (via stratification) for age, calendar period, sex, educational level at hiring, doses lagged by 10 years. There were supplementary analyses performed to assess interaction of risk with age at exposure and attained age.	3.8/5
Eldorado uranium miners and processing workers	Lane et al. 2010	2/5: External γ-ray exposure assessment via detailed job-exposure matrix for each calendar year, job type, etc, based on measurements of γ-rays. No attempt made to adjust for dose error.	4/5: Mortality assessed from start of 1/1/1950 to 31/12/1999 (so workers dying in 1932-1949 are not included in the study).	4/5: All male workers are used, alive as of 1/1/1950. Analysis adjusted for workers working < 6 months *vs* > 6 months. Analyses with exclusion of those with <6 months work gave results that were similar to those when the above adjustment was made.	0/5: None.	3/5: Poisson regression linear in external radiation dose with γ-ray doses lagged by 2 years.	2.6/5: Dosimetry via job-exposure matrix a weakness, although as errors are likely to be Berkson this should not introduce bias. Absence of lifestyle information, and lagging of dose by 2 years are a weakness.

Supplemental Material, Table S2 (cont.)

Data	Reference	Quality of dosimetry	Endpoint (mortality *vs* morbidity etc)	Selection criteria	Lifestyle circulatory disease risk factors assessed	Statistical analysis	Overall quality score/comment
3rd Analysis of UK National Registry for Radiation Workers	Muirhead et al. 2009	4/5: Individual external exposures recorded via photographic film. No attempt made to adjust for dose error.	4/5: Mortality follow-up from 1/1/1955-31/12/2001 to death or emigration or worker exceeding age 85.	5/5: Employees of Atomic Weapons Establishment, British Energy Generation and Magnox Electric Ltd (England and Scotland), British Nuclear Fuels plc (BNFL), GE Healthcare, HPA-RPD, MRC Harwell, Ministry of Defence, Organisations using the HPA personal Dosimetry Service, Rolls-Royce Submarines, Science and Technology Facilities Council, UK Atomic Energy Authority (UKAEA) who undertook radiation work on or after 1/1/1976.	1/5: Stratification by employment status (industrial *vs* non-industrial).	4/5: Poisson regression linear in external radiation dose with adjustment for age, gender, calendar period, industrial classification (industrial/non-industrial/unknown, first employer. The first ten years of follow-up after initial exposure were excluded, and all doses were lagged by 10 years.	3.6/5

Supplemental Material, Table S2 (cont.)

Data	Reference	Quality of dosimetry	Endpoint (mortality *vs* morbidity etc)	Selection criteria	Lifestyle circulatory disease risk factors assessed	Statistical analysis	Overall quality score/comment
IARC 15-country nuclear worker study	Vrijheid et al. 2007	5/5: Individual external exposures recorded via photographic film and assessments made of colon dose. Some correction for dose error.	4/5: Mortality with variable follow-up depending on facility type/cohort to 1984-2000.	4/5: Employees at one of the constituent facilities who had been employed at least 1 year, monitored for external radiation exposure, followed for non-cancer mortality and with adequate socio-economic status information (excluding the Japanese, US-Idaho National Laboratories, Canada Ontario-Hydro workforces from the 15 Country Study). Workers with more than about 10% of dose estimated to come from very high (>3000 KeV) or very low (<100 KeV) photons (X rays, γ rays), neutrons or internal radionuclides were excluded.	1/5: Socio-economic score.	5/5: Poisson regression linear and log-linear in external radiation dose adjusted (via stratification), for sex, age, calendar period, facility, duration of employment (<10 years, >10 years), socio-economic score. Interactions of dose response with gender, study cohort, facility type, age at exposure (<35, 35-50, 50+ years), time since exposure (<10, 10-20, 20+ years) and age (<60, 60-70, 70+ years) were tested via likelihood-ratio tests. Most analyses used dose lagged by 10 years.	3.8/5: exclusion of workers with substantial dose from internal radionuclides may have reduced power, although it should not have introduced bias.

Supplemental Material, Table S2 (cont.)

[a]We assess the quality of each study in a number of categories using objective criteria, as follows:

Dosimetry (out of 5)

Starting with a score of 5 points: subtract 1 point if dosimetry is not based on concurrent registry-derived records; subtract 1 point if some substantial component of dose is not assessed (e.g., neutrons); subtract 2 points if dosimetry is based on area-based assessments of exposure; subtract 1 point if no attempt is made to correct for dose error.

Endpoint (out of 5)

Starting with a score of 5 points: subtract 1 point if the follow-up is substantially incomplete (>5% of deaths/cases in cohort are lost to follow-up); subtract 1 point if follow-up is not based on local (regional), national or cohort-based registers; subtract 1 point if date of ascertainment of disease incidence/mortality may be substantially in error (> 1 year); subtract 1 point if there is no clinical review of pathology data to verify diagnosis of mortality/morbidity.

Selection criteria (out of 5)

Starting with a score of 5 points: subtract 1 point if the selection may result in omission of potentially highly exposed persons; subtract 2 points if the selection does not stringently exclude workers with missing dose records; subtract 2 points if the selection does not exclude workers working for a short time (< 6 months).

Lifestyle/circulatory disease risk factors assessed (out of 5)

Starting with a score of 5 points: subtract 1 point if there is no information on (and adjustment for) socioeconomic status; subtract 1 point if there is no information on (and adjustment for) cigarette smoking and alcohol consumption; subtract 1 point if there is no information on (and adjustment for) obesity; subtract 1 point if there is no information on (and adjustment for) diabetes; subtract 1 point if there is no information on (and adjustment for) blood pressure.

Statistical analysis (out of 5)

Starting with a score of 5 points: subtract 1 point if there is no attempt to assess interactions of dose response with age at exposure, attained age or time since exposure; subtract 1 point if the method of analysis is unclear; subtract 1 point if an inappropriate lag period is used (outside the range 5-10 years).

The mean score was obtained as the arithmetic average of these five component scores.

Table S3. Assessments of publication/selection bias using methods of Egger et al. (1997) and Steichen (1998), and bias-corrected ERR coefficients using method of Duval and Tweedie (2000).

Disease	Egger et al. publication/ selection-bias test *p*-value	Random effects excess relative risk Sv^{-1} (and 95% CI), bias-uncorrected	Random effects excess relative risk Sv^{-1} (and 95% CI), corrected using trim-and-fill method of Duval and Tweedie
Ischemic heart disease (ICD10 I20-I25)	0.322	0.10 (0.04, 0.15)	0.09 (0.02, 0.15)
Other heart disease (ICD10 I26-I52)	0.468	0.08 (-0.12, 0.28)	0.08 (-0.12, 0.28)
Cerebrovascular disease (ICD10 I60-I69)	0.692	0.21 (0.02, 0.39)	0.20 (0.02, 0.39)
Other circulatory disease (ICD10 I00-I19, I53-I59, I70-I99)	0.408	0.19 (-0.00, 0.38)	0.16 (-0.03, 0.35)

Table S4. Sensitivity of Risk Estimates to Study Exclusion

Study Excluded	Fixed-effect estimate of ERR per Sv (95% CI)	Random-effect estimate of ERR per Sv (95% CI)	1-sided *P* (fixed effect / random effect)	Heterogeneity, *P*
Ischemic Heart Disease (OCD10 I20-I25)				
Yamada et al. 2004	0.11 (0.05 to 0.17)	0.11 (0.05 to 0.17)	<0.001 / <0.001	0.396
Ivanov et al. 2006	0.09 (0.04 to 0.14)	0.09 (0.04 to 0.14)	<0.001 / <0.001	0.635
Vrijheid et al. 2007	0.10 (0.05 to 0.15)	0.10 (0.03 to 0.16)	<0.001 / 0.002	0.312
Muirhead et al. 2009	0.09 (0.04 to 0.14)	0.09 (0.04 to 0.15)	<0.001 / <0.001	0.396
Azizova et al. 2010a[a]	0.07 (-0.01 to 0.14)	0.07 (-0.01 to 0.15)	0.035 / 0.037	0.398
Shimizu et al. 2010	0.11 (0.06 to 0.17)	0.11 (0.06 to 0.17)	<0.001 / <0.001	0.480
Laurent et al. 2010	0.10 (0.05 to 0.15)	0.09 (0.04 to 0.15)	<0.001 / <0.001	0.362
Lane et al. 2010	0.10 (0.04 to 0.15)	0.09 (0.03 to 0.16)	<0.001 / 0.002	0.310
None (all studies)	0.10 (0.05 to 0.15)	0.10 (0.04 to 0.15)	<0.001 / <0.001	0.408
Non-Ischemic Heart Disease (ICD10 I26-I52)				
Ivanov et al. 2006	0.14 (0.01 to 0.27)	0.13 (-0.05 to 0.30)	0.016 / 0.076	0.263
Vrijheid et al. 2007[b]	0.12 (-0.01 to 0.25)	0.06 (-0.16 to 0.29)	0.031 / 0.289	0.098
Shimizu et al. 2010[c]	-0.26 (-0.80 to 0.28)	-0.26 (-0.80 to 0.28)	0.824 / 0.824	0.928
None (all studies)	0.12 (-0.01 to 0.25)	0.08 (-0.12 to 0.28)	0.031 / 0.222	0.199

Supplemental Material, Table S4 (cont.)

Study Excluded	Fixed-effect estimate of ERR per Sv (95% CI)	Random-effect estimate of ERR per Sv (95% CI)	1-sided *P* (fixed effect / random effect)	Heterogeneity, *P*
Cerebrovascular Disease (ICD10 I60-I69)				
Yamada et al. 2004	0.21 (0.16 to 0.27)	0.24 (0.01 to 0.46)	<0.001 / 0.019	<0.001
Ivanov et al. 2006	0.19 (0.14 to 0.25)	0.17 (-0.03 to 0.37)	<0.001 / 0.048	<0.001
Kreuzer et al. 2006	0.20 (0.14 to 0.25)	0.21 (0.02 to 0.40)	<0.001 / 0.015	<0.001
Vrijheid et al. 2007	0.20 (0.14 to 0.25)	0.20 (0.01 to 0.38)	<0.001 / 0.017	<0.001
Muirhead et al. 2009	0.20 (0.14 to 0.25)	0.21 (0.02 to 0.40)	<0.001 / 0.017	<0.001
Azizova et al. 2010b[d]	0.12 (0.06 to 0.18)	0.12 (0.02 to 0.23)	<0.001 / 0.010	0.310
Shimizu et al. 2010	0.31 (0.23 to 0.40)	0.22 (-0.02 to 0.46)	<0.001 / 0.034	0.002
Laurent et al. 2010	0.20 (0.14 to 0.25)	0.20 (0.02 to 0.39)	<0.001 / 0.013	<0.001
Lane et al. 2010	0.20 (0.15 to 0.26)	0.24 (0.06 to 0.43)	<0.001 / 0.005	<0.001
None (all studies)	0.20 (0.14 to 0.25)	0.21 (0.02 to 0.39)	<0.001 / 0.014	<0.001

Supplemental Material, Table S4 (cont.)

Study Excluded	Fixed-effect estimate of ERR per Sv (95% CI)	Random-effect estimate of ERR per Sv (95% CI)	1-sided *P* (fixed effect / random effect)	Heterogeneity, *P*
Circulatory Disease Apart from Heart Disease and Cerebrovascular Disease (ICD10 I00-I19, I53-I59, I70-I99)				
Yamada et al. 2004[e]	0.48 (0.36 to 0.59)	0.35 (0.02 to 0.68)	<0.001 / 0.018	0.003
Ivanov et al. 2006[f]	0.09 (0.05 to 0.14)	0.22 (-0.02 to 0.45)	<0.001 / 0.035	<0.001
Shimizu et al. 2010[g]	0.04 (-0.01 to 0.08)	0.04 (-0.04 to 0.11)	0.052 / 0.171	0.219
None (all studies)	0.10 (0.05 to 0.14)	0.19 (0.00 to 0.38)	<0.001 / 0.026	<0.001

[a]Analysis based on morbidity from ischemic heart disease, with a 10-year lag.
[b]Analysis based on mortality from heart failure.
[c]Analysis based on mortality from heart failure and other heart disease.
[d]Analysis based on morbidity from cerebrovascular disease, with a 10-year lag.
[e]Analysis based on morbidity from hypertension, hypertensive heart disease and aortic aneurysm.
[f]Analysis based on morbidity from hypertension, disease of arteries, arterioles and capillaries, veins, lymphatic vessels and lymph nodes.
[g]Analysis based on mortality from rheumatic heart disease and circulatory disease apart from heart disease and cerebrovascular disease.

Table S5. Models fitted individually to stroke, heart disease and all other circulatory disease in the Japanese atomic-bomb survivor Life Span Study mortality data

No	Relative risk model[a]	Stroke		Heart disease		Other circulatory disease	
		Deviance (df)	p-value[b]	Deviance (df)	p-value[b]	Deviance (df)	p-value[b]
1	Null	10500.46 (26026)	-	11677.60 (26026)	-	4470.68 (26026)	-
2	1 + *ERR D*	10487.90 (26025)	0.000	11648.48 (26025)	0.000	4369.26 (26025)	0.000
3	1 + *ERR D* exp[α (e - 30)]	10480.63 (26024)	0.007	11647.74 (26024)	0.391	4336.58 (26024)	0.000
4	1 + *ERR D* exp[α (e - 30) + β (y - 30)]	10480.31 (26023)	0.573	11647.60 (26023)	0.705	4336.46 (26023)	0.726
5	1 + *ERR D* exp[α ($e + y$ - 60)]	10484.74 (26024)	0.076[c]	11647.81 (26024)	0.412[c]	4344.85 (26024)	0.000[c]
6	1 + *ERR D* exp[β (y - 30)]	10486.72 (26024)	0.278[d]	11648.47 (26024)	0.938[d]	4366.40 (26024)	0.091[d]
7	1 + *ERR D* exp[α (e - 30) + γ $1_{\text{sex=female}}$]	10480.61 (26023)	0.903[e]	11642.50 (26023)	0.022[e]	4336.58 (26023)	1.000[e]
8	1 + *ERR* ($D + \beta D^2$) exp[α (e - 30)]	10478.80 (26023)	0.177[f]	11647.72 (26023)	0.877[f]	4336.25 (26023)	0.560[f]

[a]y = years since exposure, e = years of age at exposure
[b]unless otherwise stated, all p-values indicate the improvement in fit over the model on the previous line.
[c]improvement in fit of model 5 *vs* model 2.
[d]improvement in fit of model 6 *vs* model 2.
[e]improvement in fit of model 7 *vs* model 3.
[f]improvement in fit of model 8 *vs* model 3.

Table S6. Models jointly fitted to stroke, heart disease and all other circulatory disease in the Japanese atomic-bomb survivor Life Span Study mortality data (optimal model shown in boldface)

No	Relative risk model[a]	Deviance (df)	p-value[b]	ERR (stroke) (+95% CI)	ERR (heart disease) (+95% CI)	ERR (other CVD) (+95% CI)	Age at exposure (α)(stroke) (+95% CI)	Age at exposure (α)(heart disease)(+95% CI)	Age at exposure (α)(other CVD)(+95% CI)	Years since exposure (β)(stroke)(+95% CI)	Years since exposure (β)(heart disease)(+95% CI)	Years since exposure (β)(other CVD)(+95% CI)
1	Null	26648.74 (78078)	-	-	-	-	-	-	-	-	-	-
2	$1 + ERR\ D$	26546.58 (78077)	<0.001	0.22 (0.18, 0.27)	0.22 (0.18, 0.27)	0.22 (0.18, 0.27)	-	-	-	-	-	-
3	$1 + ERR_i\ D$	26505.64 (78075)	<0.001	0.12 (0.05, 0.19)	0.18 (0.11, 0.25)	0.58 (0.45, 0.72)	-	-	-	-	-	-
4	$1 + ERR_i\ D \exp[\alpha\ (e - 30)]$	26470.83 (78074)	<0.001	0.17 (0.09, 0.26)	0.17 (0.09, 0.25)	0.60 (0.46, 0.75)	-0.044 (-0.061, -0.030)	-0.044 (-0.061, -0.030)	-0.044 (-0.061, -0.030)	-	-	-
**5**	$\boldsymbol{1 + ERR_i\ D \exp[\alpha_i\ (e - 30)]}$	**26464.95 (78072)**	**0.053**	**0.17 (0.07, 0.26)**	**0.19 (0.12, 0.28)**	**0.56 (0.41, 0.72)**	**-0.050 (-0.099, -0.015)**	**-0.012 (-0.041, 0.018)**	**-0.055 (-0.075, -0.036)**	-	-	-
6	$1 + ERR_i\ D \exp[\alpha\ (e + y - 60)]$	26484.45 (78074)	<0.001[c]	0.18 (0.09, 0.28)	0.22 (0.13, 0.32)	1.05 (0.77, 1.38)	-0.033 (-0.046, -0.019)	-0.033 (-0.046, -0.019)	-0.033 (-0.046, -0.019)	-	-	-
7	$1 + ERR_i\ D \exp[\alpha_i\ (e + y - 60)]$	26477.40 (78072)	0.029	0.18 (0.09, 0.29)	0.20 (0.11, 0.30)	1.24 (0.91, 1.63)	-0.040 (-0.084, 0.005)	-0.010 (-0.032, 0.016)	-0.048 (-0.067, -0.030)	-	-	-
8	$1 + ERR_i\ D \exp[\beta\ (y - 30)]$	26503.44 (78074)	0.138[d]	0.12 (0.05, 0.19)	0.15 (0.09, 0.23)	0.46 (0.29, 0.67)	-		-	0.015 (-0.005, 0.037)	0.015 (-0.005, 0.037)	0.015 (-0.005, 0.037)
9	$1 + ERR_i\ D \exp[\beta_i\ (y - 30)]$	26501.59 (78072)	0.397	0.11 (0.03, 0.19)	0.18 (0.10, 0.26)	0.36 (0.17, 0.65)	-		-	0.022 (-0.020, 0.082)	-0.001 (-0.030, 0.031)	0.028 (-0.004, 0.064)
10	$1 + ERR_i\ D \exp[\alpha\ (e - 30) + \beta\ (y - 30)]$	26470.57 (78073)	0.609[e]	0.18 (0.09, 0.29)	0.18 (0.09, 0.28)	0.65 (0.42, 0.96)	-0.045 (-0.062, -0.030)	-0.045 (-0.062, -0.030)	-0.045 (-0.062, -0.030)	-0.005 (-0.025, 0.016)	-0.005 (-0.025, 0.016)	-0.005 (-0.025, 0.016)
11	$1 + ERR_i\ D \exp[\alpha_i\ (e - 30) + \beta_i\ (y - 30)]$	26464.37 (78069)	0.185	0.19 (0.07, 0.30)	0.20 (0.11, 0.30)	0.62 (0.31, 1.10)	-0.055 (-0.104, -0.014)	-0.014 (-0.042, 0.018)	-0.056 (-0.077, -0.036)	-0.012 (-0.056, 0.039)	-0.006 (-0.035, 0.027)	-0.006 (-0.040, 0.029)

[a] y = years since exposure, e = years of age at exposure, index i refers to the disease endpoint (stroke, heart disease, other circulatory disease)

[b] unless otherwise stated, all p-values indicate the improvement in fit over the model on the previous line.

[c] improvement in fit of model 6 *vs* model 3.

[d] improvement in fit of model 8 *vs* model 3.

[e] improvement in fit of model 10 *vs* model 4.

Table S7. Estimated Excess Relative Risks of Circulatory Disease in Radiotherapeutically Treated Groups. (Adapted from Little *et al.* (2008; 2010)). All data are in relation to underlying cause of death, unless otherwise indicated.

Data	Reference	Average heart/brain dose (range) (Sv)	Numbers in cohort (person years follow-up)	Endpoint (mortality unless otherwise indicated)	Excess relative risk Sv^{-1} (and 95% CI)
French-UK childhood cancer study	Tukenova et al. 2010	11.1[a] (<1 – >15))	4122[b] (n.a.)	All cardiovascular disease	0.6 (0.2, 2.5)
US Childhood Cancer Survivor Study	Mulrooney et al. 2009	n.a. (<5 – > 35)	14,358 (n.a.)	Congestive heart disease morbidity<br>Myocardial infarction morbidity<br>Pericardial disease morbidity<br>Valvular disease morbidity	0.05 (0.02, 0.09)[c]<br>0.04 (-0.02, 0.10)[c]<br>0.05 (-0.01, 0.11)[c]<br>0.07 (-0.02, 0.16)[c]
Peptic ulcer study	Little et al. 2012	1.01 (0.0 – 6.20)[d]	3600 (76,571.7)	Coronary heart disease (ICD8 410-414)<br>Stroke (ICD8 430-438)<br>All other circulatory disease<br>All circulatory disease (ICD8 390-459)	0.102 (0.039, 0.174)[d]<br>0.028 (-0.085, 0.186)[d]<br>0.050 (-0.053, 0.194)[d]<br>0.082 (0.031, 0.140
Ankylosing spondylitis	Darby et al. 1987	0.14 (0.0 - 4.80)[e]<br>2.49 (0.0 – 17.28)[d]	14,106 (183,749)	Stroke (ICD7 430-434)<br>Other circulatory disease (ICD7 400-429, 435-468)	-2.43 (-4.29, 0.71)[e f]<br>-0.01 (-0.12, 0.13)[f d]
TB fluoroscopy	Davis et al. 1989	0.84[g] (n.a.)	13,385 (331,006)	All circulatory disease (ICD8 390-458)	-0.11 (-0.20, -0.01)[g]

[a]Mean heart dose to 21 persons who died of cardiovascular disease.
[b]5-year survivors.
[c]Estimate derived by fitting linear model (using method of Little *et al.* (2008)) by weighted least squares, applied to aggregate data given in Table 4 of Mulrooney *et al.* (2009), assuming average cardiac doses of 0, 2.5, 10, 25, 40 Gy to the cardiac dose groups 0, 0-5, 5-15, 15-35 and >35 Gy.
[d]Based on heart dose.
[e]Based on brain dose.
[f]Based on ERR and 95% CI given in reference (McGale and Darby 2005),combined with the median organ dose estimate of reference. (Lewis et al. 1988)
[g]Based on lung dose.

Reference List

Azizova TV, Muirhead CR, Druzhinina MB, Grigoryeva ES, Vlasenko EV, Sumina MV, et al. 2010a. Cardiovascular diseases in the cohort of workers first employed at Mayak PA in 1948-1958. Radiat Res 174:155-168.

-----. 2010b. Cerebrovascular Diseases in the Cohort of Workers First Employed at Mayak PA in 1948-1958. Radiat Res 174:851-864.

Darby SC, Doll R, Gill SK, Smith PG. 1987. Long term mortality after a single treatment course with X-rays in patients treated for ankylosing spondylitis. Br J Cancer 55:179-190.

Davis FG, Boice JD, Jr., Hrubec Z, Monson RR. 1989. Cancer mortality in a radiation-exposed cohort of Massachusetts tuberculosis patients. Cancer Res 49:6130-6136.

Egger M, Davey SG, Schneider M, Minder C. 1997. Bias in meta-analysis detected by a simple, graphical test. BMJ 315:629-634.

Ivanov VK, Maksioutov MA, Chekin SY, Petrov AV, Biryukov AP, Kruglova ZG, et al. 2006. The risk of radiation-induced cerebrovascular disease in Chernobyl emergency workers. Health Phys 90:199-207.

Kreuzer M, Kreisheimer M, Kandel M, Schnelzer M, Tschense A, Grosche B. 2006. Mortality from cardiovascular diseases in the German uranium miners cohort study, 1946-1998. Radiat Environ Biophys 45:159-166.

Lane RS, Frost SE, Howe GR, Zablotska LB. 2010. Mortality (1950-1999) and cancer incidence (1969-1999) in the cohort of Eldorado uranium workers. Radiat Res 174:773-785.

Laurent O, Metz-Flamant C, Rogel A, Hubert D, Riedel A, Garcier Y, et al. 2010. Relationship between occupational exposure to ionizing radiation and mortality at the French electricity company, period 1961-2003. Int Arch Occup Environ Health 83:935-944.

Lewis CA, Smith PG, Stratton IM, Darby SC, Doll R. 1988. Estimated radiation doses to different organs among patients treated for ankylosing spondylitis with a single course of X rays. Br J Radiol 61:212-220.

Little MP, Kleinerman RA, Stovall M, Smith SA, Mabuchi K. 2012. Analysis of Dose Response for Circulatory Disease After Radiotherapy for Benign Disease. Int J Radiat Oncol Biol Phys in press doi:10.1016/j.ijrobp.2012.01.053.

Little MP, Tawn EJ, Tzoulaki I, Wakeford R, Hildebrandt G, Paris F, et al. 2008. A systematic review of epidemiological associations between low and moderate doses of ionizing radiation and late cardiovascular effects, and their possible mechanisms. Radiat Res 169:99-109.

-----. 2010. Review and meta-analysis of epidemiological associations between low/moderate doses of ionizing radiation and circulatory disease risks, and their possible mechanisms. Radiat Environ Biophys 49:139-153.

McCullagh P, Nelder JA. 1989. Generalized linear models. 2nd edition. In: Monographs on statistics and applied probability 37 Boca Raton, FL:Chapman and Hall/CRC, 1-526.

McGale P, Darby SC. 2005. Low doses of ionizing radiation and circulatory diseases: a systematic review of the published epidemiological evidence. Radiat Res 163:247-257.

Muirhead CR, O'Hagan JA, Haylock RG, Phillipson MA, Willcock T, Berridge GL, et al. 2009. Mortality and cancer incidence following occupational radiation exposure: third analysis of the National Registry for Radiation Workers. Br J Cancer 100:206-212.

Mulrooney DA, Yeazel MW, Kawashima T, Mertens AC, Mitby P, Stovall M, et al. 2009. Cardiac outcomes in a cohort of adult survivors of childhood and adolescent cancer: retrospective analysis of the Childhood Cancer Survivor Study cohort. BMJ 339:b4606.

Shimizu Y, Kodama K, Nishi N, Kasagi F, Suyama A, Soda M, et al. 2010. Radiation exposure and circulatory disease risk: Hiroshima and Nagasaki atomic bomb survivor data, 1950-2003. BMJ 340:b5349.

Steichen TJ. 1998. Tests for publication bias in meta-analysis. In: sbe19 College Station, Texas, USA:Stata Press, 9-15.

Stroup DF, Berlin JA, Morton SC, Olkin I, Williamson GD, Rennie D, et al. 2000. Meta-analysis of observational studies in epidemiology: a proposal for reporting. Meta-analysis Of Observational Studies in Epidemiology (MOOSE) group. JAMA 283:2008-2012.

Talbott EO, Youk AO, McHugh-Pemu KP, Zborowski JV. 2003. Long-term follow-up of the residents of the Three Mile Island accident area: 1979-1998. Environ Health Perspect 111:341-348.

Tukenova M, Guibout C, Oberlin O, Doyon F, Mousannif A, Haddy N, et al. 2010. Role of cancer treatment in long-term overall and cardiovascular mortality after childhood cancer. J Clin Oncol 28:1308-1315.

Vrijheid M, Cardis E, Ashmore P, Auvinen A, Bae JM, Engels H, et al. 2007. Mortality from diseases other than cancer following low doses of ionizing radiation: results from the 15-Country Study of nuclear industry workers. Int J Epidemiol 36:1126-1135.

Yamada M, Wong FL, Fujiwara S, Akahoshi M, Suzuki G. 2004. Noncancer disease incidence in atomic bomb survivors, 1958-1998. Radiat Res 161:622-632.
